# Identification of novel cerebrospinal fluid biomarker candidates for dementia with Lewy bodies: a proteomic approach

**DOI:** 10.1186/s13024-020-00388-2

**Published:** 2020-06-18

**Authors:** Inger van Steenoven, Marleen J. A. Koel-Simmelink, Leonie J. M. Vergouw, Betty M. Tijms, Sander R. Piersma, Thang V. Pham, Claire Bridel, Gian-Luca Ferri, Cristina Cocco, Barbara Noli, Paul F. Worley, Mei-Fang Xiao, Desheng Xu, Patrick Oeckl, Markus Otto, Wiesje M. van der Flier, Frank Jan de Jong, Connie R. Jimenez, Afina W. Lemstra, Charlotte E. Teunissen

**Affiliations:** 1 Alzheimer Center Amsterdam, Department of Neurology, Amsterdam Neuroscience, Vrije Universiteit Amsterdam, Amsterdam UMC, De Boelelaan 1118, 1081 HV Amsterdam, The Netherlands; 2 Neurochemistry Laboratory and Biobank, Department of Clinical Chemistry, Amsterdam Neuroscience, Vrije Universiteit Amsterdam, Amsterdam UMC, Amsterdam, The Netherlands; 3 Alzheimer Center Erasmus MC, Department of Neurology, Erasmus Medical Center, Rotterdam, The Netherlands; 4 OncoProteomics Laboratory, Department of Medical Oncology, Vrije Universiteit Amsterdam, Amsterdam UMC, Amsterdam, The Netherlands; 5 NEF-laboratory, Department of Biomedical Sciences, University of Cagliari, Monserrato, Italy; 6 Solomon H. Snyder Department of Neuroscience, Johns Hopkins University School of Medicine, Baltimore, USA; 7 Department of Neurology, Johns Hopkins University School of Medicine, Baltimore, USA; 8 Department of Neurology, Ulm University Hospital, Ulm, Germany; 9 Department of Epidemiology and Biostatistics, Amsterdam Neuroscience, Vrije Universiteit Amsterdam, Amsterdam UMC, Amsterdam, The Netherlands

**Keywords:** Biomarkers, Cerebrospinal fluid, Dementia with Lewy bodies, Lewy body dementia, Proteomics

## Abstract

**Background:**

Diagnosis of dementia with Lewy bodies (DLB) is challenging, largely due to a lack of diagnostic tools. Cerebrospinal fluid (CSF) biomarkers have been proven useful in Alzheimer’s disease (AD) diagnosis. Here, we aimed to identify novel CSF biomarkers for DLB using a high-throughput proteomic approach.

**Methods:**

We applied liquid chromatography/tandem mass spectrometry with label-free quantification to identify biomarker candidates to individual CSF samples from a well-characterized cohort comprising patients with DLB (*n* = 20) and controls (*n* = 20). Validation was performed using (1) the identical proteomic workflow in an independent cohort (*n* = 30), (2) proteomic data from patients with related neurodegenerative diseases (*n* = 149) and (3) orthogonal techniques in an extended cohort consisting of DLB patients and controls (*n* = 76). Additionally, we utilized random forest analysis to identify the subset of candidate markers that best distinguished DLB from all other groups.

**Results:**

In total, we identified 1995 proteins. In the discovery cohort, 69 proteins were differentially expressed in DLB compared to controls (*p* < 0.05). Independent cohort replication confirmed VGF, SCG2, NPTX2, NPTXR, PDYN and PCSK1N as candidate biomarkers for DLB. The downregulation of the candidate biomarkers was somewhat more pronounced in DLB in comparison with related neurodegenerative diseases. Using random forest analysis, we identified a panel of VGF, SCG2 and PDYN to best differentiate between DLB and other clinical groups (accuracy: 0.82 (95%CI: 0.75–0.89)). Moreover, we confirmed the decrease of VGF and NPTX2 in DLB by ELISA and SRM methods. Low CSF levels of all biomarker candidates, except PCSK1N, were associated with more pronounced cognitive decline (0.37 < r < 0.56, all *p* < 0.01).

**Conclusion:**

We identified and validated six novel CSF biomarkers for DLB. These biomarkers, particularly when used as a panel, show promise to improve diagnostic accuracy and strengthen the importance of synaptic dysfunction in the pathophysiology of DLB.

## Background

Dementia with Lewy Bodies (DLB) is a common cause of dementia in the elderly, accounting for up to 20% of dementia cases [1]. Clinical hallmarks of DLB are cognitive decline accompanied by parkinsonism, visual hallucinations, fluctuating cognition and rapid eye movement (REM) sleep behavior disorder (RBD) [2]. Diagnosis of DLB during life is based on clinical diagnostic consensus criteria [2], but a definite diagnosis of DLB requires post-mortem defined presence of Lewy bodies and Lewy neurites diffusely distributed throughout the brain [2, 3]. Diagnosing DLB during life is challenging due to highly variable clinical manifestation and overlap in signs, symptoms and pathology with both Alzheimer’s disease (AD) and Parkinson’s disease (PD). There is thus a strong need for biomarkers supporting accurate and timely diagnosis of DLB.

Cerebrospinal fluid (CSF) is the best matrix to identify novel biomarkers for central nervous system disorders, due to its direct contact with the brain parenchyma and mirroring biochemical alterations occurring within the brain [4, 5]. CSF biomarkers have been proven useful in AD, where a typical CSF profile of decreased levels of amyloid-β 1–42 (Aβ_1–42_) combined with increased levels of total and phosphorylated tau (t-tau, p-tau) protein levels supports the diagnosis of AD [6]. So far, no such diagnostic biomarkers are available for DLB. CSF biomarkers for α-synuclein seem promising [7–9], but are still not sensitive and specific enough to function as single diagnostic biomarkers.

Mass spectrometry-based proteomics has emerged as an useful approach for unbiased candidate biomarker discovery in biofluids [10, 11]. So far, only few proteomic studies have been performed for DLB, albeit in small and clinically heterogeneous cohorts, and results have not yet been validated [12–14].

Here, we aimed to identify novel candidate proteins in CSF of DLB patients in a relatively large, well-characterized discovery cohort (20 DLB patients and 20 controls) using a state-of-the-art mass spectrometry workflow. We next thoroughly validated the results by (1) the same proteomic workflow in an independent cohort (*n* = 30), (2) comparison of identified biomarkers values in related neurodegenerative diseases (*n* = 149) and (3) enzyme-linked immunosorbent assays (ELISA) and selected reaction monitoring (SRM) for the most represented candidate biomarkers in an extended cohort (*n* = 76).

## Methods

### Patient selection

DLB patients and controls enrolled in the current study were selected from the Amsterdam Dementia Cohort and the Erasmus Medical Center. All subjects underwent extensive clinical examination including physical and neurological examination, neuropsychological assessment, electroencephalogram, structural brain imaging and laboratory tests [15]. Additional diagnostic tests, such as ^123^I[FP-CIT] single photon emission computed tomography (DaT-SPECT) were performed by indication. Diagnoses were made by consensus in a multidisciplinary meeting according to standard diagnostic criteria. Probable DLB was diagnosed according to the 2005 clinical consensus criteria [16]. All patients also fulfilled novel consensus criteria [2]. Controls were individuals who presented at the memory clinic with cognitive complaints, but no abnormalities on clinical or cognitive testing were observed and criteria for mild cognitive impairment, dementia or other medical conditions associated with cognitive complaints were not met. Furthermore, all controls had normal AD biomarker levels in CSF [17], and preserved normal cognitive function on neuropsychological testing for at least two years after first presentation at the memory clinic. The study was performed according to the ethical principles of the Declaration of Helsinki and was approved by the local ethics committees. Written informed consent was obtained from all subjects.

#### Phase 1: discovery

##### Cohort 1

For the biomarker discovery phase, 20 DLB patients and age- and sex-matched controls were selected from the Amsterdam Dementia Cohort according to the criteria described above. In addition, DLB patients in cohort 1 fulfilled the following additional inclusion criteria: (1) DaT-SPECT scan showing presynaptic dopaminergic deficits and (2) normal AD biomarker levels in the CSF [17].

#### Phase 2: proteomics validation

##### Cohort 2

A second cohort consisted of an independent set of 17 DLB patients and 13 age- and sex-matched controls selected from the Amsterdam Dementia Cohort (*n* = 27) and the Erasmus Medical Center (*n* = 3) was used for validation using an identical proteomics workflow. The DLB patients in cohort 2 had less stringent inclusion criteria, namely DLB patients were not selected on the basis of normal CSF AD biomarker levels and a DaT-SPECT scan was not required.

#### Phase 3: validation of candidate biomarkers

##### Cohort 3A

For the validation of the identified candidate biomarkers in related neurodegenerative diseases, we analyzed proteomic data previously generated in 20 patients with AD and 20 patients with frontotemporal dementia (FTD) as part of a parallel study (PRODIA Memorabel Project). In addition, proteomic data from 109 PD patients were provided by the Fox Investigation for New Discovery of Biomarkers (“BioFIND”) database (http://biofind.loni.usc.edu/) [18].

##### Cohort 3B

A subset of the identified candidate biomarkers was validated by orthogonal analytical techniques in CSF samples from DLB patients and controls. Cohort 3B consisted of 48 DLB patients and 28 controls selected from the Amsterdam Dementia Cohort. Cohort 3B was not completely independent from cohort 1 and 2, such that 15 controls and 18 DLB patients were overlapping between cohort 3B and cohort 1 and 3 controls and 6 DLB patients were overlapping between cohort 3B and cohort 2. DLB patients in cohort 3B fulfilled similar inclusion criteria as DLB patients in cohort 2.

### CSF sample collection and storage

In line with international biobanking guidelines [19], CSF was obtained by lumbar puncture between the L3/L4, L4/L5 or L5/S1 intervertebral space using a 25-gauge needle and collected in 10 mL polypropylene tubes (Starstedt, Nümbrecht, Germany). Part of the CSF was used for basic CSF analysis, and levels of Aß_1–42_, total tau and p-tau were measured with commercially ELISA’s (Innotest®, Fujirebio, Gent, Belgium). The remaining CSF was centrifuged at 1800 g at 4 °C for 10 min, aliquoted in polypropylene tubes of 0.5 mL and stored at − 80 °C [19] until further analysis.

### Biomarker discovery analysis and validation

**Fig. 1:**
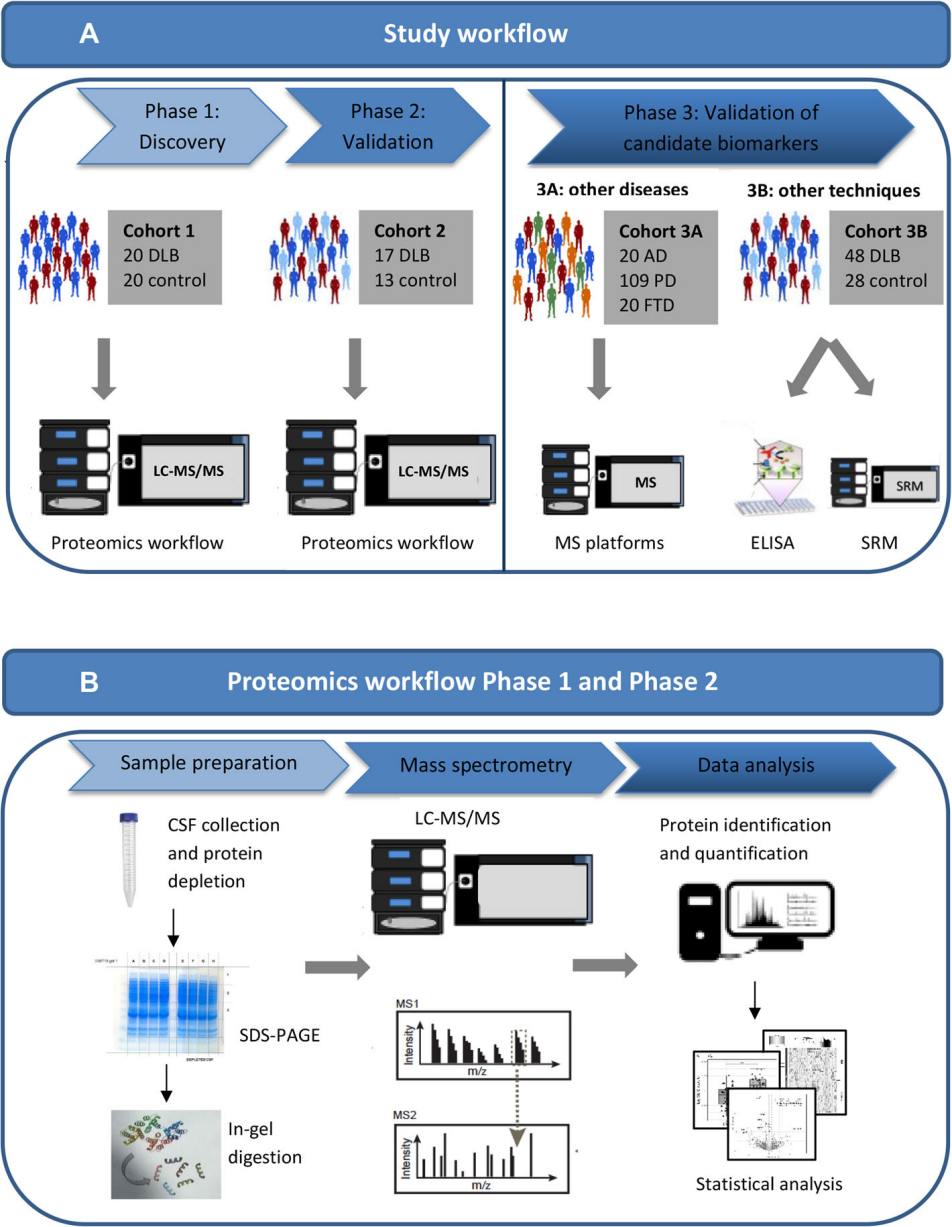
Graphical summary of the workflow used to identify novel CSF biomarkers for DLB. **a** Graphical summary of study workflow. In short, CSF samples from cohort 1 were evaluated using a high-throughput proteomic workflow. The CSF proteome from DLB patients was compared with that of cognitively normal individuals. Validation was performed in an independent validation cohort (cohort 2) using an identical proteomic workflow. Proteins that were significantly altered in abundance in both cohort 1 and cohort 2 were indicated as candidate biomarkers. Levels of the identified candidate biomarkers in DLB patients were compared with the levels of the identified candidate biomarkers as quantified with mass spectrometry in related neurodegenerative diseases (cohort 3A). For a subset of the candidate biomarkers validation was performed using orthogonal methods (ELISA and SRM) in cohort 3B. **b** Graphical summary of the proteomic workflow. We applied an in-depth proteomic workflow, including abundant protein depletion, protein fractionation prior to nanoLC-MS/MS analysis and label-free protein quantification on CSF samples from DLB patients and controls in cohort 1 and 2

The workflow for mass-spectrometry biomarker discovery analysis and validation is summarized in Fig. 1.

#### CSF sample preparation and gel electrophoresis

CSF samples were coded and analyzed in a blinded fashion. The depletion of the top-14 high abundant proteins, i.e. albumin, IgG, antitrypsin, IgA, transferrin, haptoglobulin, fibrinogen, α2-macroglobulin, α1-acid glycoprotein, IgM, apolipoprotein AI, apolipoprotein AII, complement C3 and transthyretin, was performed as previously reported [20]. Depleted CSF was further concentrated using 3kDA filters (Millipore, Billericam, CA, USA) prior to loading the whole depleted CSF fraction on 1-D gradient gels from Invitrogen (Carsbad, CA, USA; NuPAGE 4–12% Bis-Tris gel., 1.5 mm × 10 wells). SDS-PAGE gels were stained overnight with Coomassie brilliant blue R250 (Pierce, Rockford, IL, USA). To minimize inter-run variability, each gel contained four patients and four controls in an alternating order.

#### NanoLC-MS/MS analysis

Before NanoLC-MS/MS analysis, separated proteins were in-gel digested as previously described [21]. Peptides were separated by an Ultimate 3000 nanoLC system (Dionex LC-Packings, Amsterdam, The Netherlands) equipped with a 20 cm × 75 μm ID fused silica column custom packed with 3 μm 120 Å ReproSil Pur C18 aqua (Dr Maisch GMBH, Ammerbuch-Entringen, Germany). After injection, peptides were trapped at 6 μL/min on a 10 mm × 100 μm ID 5 μm 120 Å ReproSil Pur C18 aqua at 2% buffer B (buffer A: 0.05% FC in MilliQ; buffer B: 80% ACN + 0.05% FC in MilliQ). Peptides were separated at 300 nl/min in a 10–40% buffer B gradient in 60 min. Eluting peptides were ionized at a potential of + 2 kVA and injected in a QExactive mass spectrometer (Thermo Fisher, Bremen, Germany). Intact masses were measured at resolution 70.000 (at m/z 200) in the Orbitrap using an AGC target value of 3 × 10^6^ charges. The top 10 peptide signals (charge-states 2^+^ and higher) were submitted to MS/MS in the higher-energy collision cell (4 amu isolation width, 25% normalized collision energy). MS/MS spectra were acquired at resolution 17.500 (at m/z 200) in the Orbitrap using an AGC target value of 2 × 10^5^ charges and an underfill ratio of 0.1%. Dynamic exclusion was applied with a repeat count of 1 and an exclusion time of 30 s.

#### Protein identification and quantification

MS/MS spectra were searched against the Swissprot human 2018 reference proteome using MaxQuant 1.6.0.16 [22]. Enzyme specificity was set to trypsin and up to two missed cleavages were allowed. Cysteine carboxamidomethylation was set as fixed modification and methionine oxidation and N-terminal acetylation as variable modifications. Peptide precursor ions and fragment ions were searched with maximum mass deviation of 4.5 ppm and 20 ppm, respectively. All identifications were filtered at a false discovery rate (FDR) of 1% using the decoy database strategy. Protein abundance was quantified by MS-signal intensity of the area under the chromatographic peak of the peptide precursor ion.

#### Statistical analysis

To identify differentially expressed proteins among the DLB and control groups raw intensities were processed using the label-free quantification (LFQ) algorithm in MaxQuant and MaxLFQ intensities were obtained [23]. Missing values were imputed from a normal distribution centered at the minimal intensity and a variance equal to the average variance across all proteins. Hierarchical clustering was performed on log10 normalized expression using the Euclidean distance and complete linkage for both sample clustering and protein clustering. Heatmaps were generated to visualize the normalized to zero mean unit variance (z-scores) for individual proteins. Differential expression analysis was performed with the limma package available from the Bioconductor package [24].

### Proteomic analysis in CSF samples of AD, FTD and PD patients

We obtained proteomic data from CSF samples from AD and FTD patients that were generated in previous, published [25] and yet unpublished studies from our group. The proteomic analyses were performed using a similar workflow as the proteomic workflow described above. As part of a parallel study (PRODIA Memorabel Project), the generated raw proteomic data of AD, FTD and DLB patients included in cohort 1 and cohort 2 were reanalyzed against the same reference database (Swissprot human 2018 reference proteome using MaxQuant 1.6.0.16). In addition, we obtained CSF proteomic data provided by the BioFind database. CSF samples from PD patients were analyzed using state-of-the-art DEEP SEQ mass spectrometry technology [26]. The PD data were searched against a human protein database (uniprot.org) with Mascot.

### Orthogonal methods for validation of candidate biomarkers

For Neurosecretory protein VGF (VGF) and Neuronal pentraxin 2 (NPTX2), we performed additional validation experiments using orthogonal techniques (i.e., ELISA and SRM) in cohort 3B. For VGF, CSF levels of the VGF_373–417_ peptide were measured by quantitative competitive ELISA [27–29] and by SRM (see detailed description below). CSF levels of NPTX2 were detected using a quantitative sandwich ELISA, as previously described [30].

#### ELISA analysis of VGF

The human VGF_373–417_ ELISA was carried out as described [27, 28], on the basis of the corresponding rat VGF_375–420_ assay [29]. A synthetic peptide corresponding to human VGF_373–382_ (conjugated with keyhole limpet haemocyanin via an additional C-terminal Cysteine), was used for rabbit immunizations. Briefly, plates were coated with the corresponding synthetic peptide in carbonate/bicarbonate buffer (pH 9.6), blocked in PBS-Tween 20 containing normal donkey serum (90 mL/L), aprotinin (20 nmol/L) and EDTA (1 g/L), and incubated with a mixture of primary antibody (in the same medium), and serial dilutions of either standard peptide, or samples. For the standard curve, a range of concentrations (50 nmol/L to 50 fmol/L) of either VGF_373–382_ or VGF_373–417_ (GGEE-45) synthetic peptide was used. The latter was identified as a natural peptide in human CSF [31], hence was used as “full length” reference. After primary incubation, plates were washed, treated with biotinylated secondary antibodies (Jackson, West Grove, PA, USA), streptavidin-peroxidase conjugate (Biospa, Milan, Italy), and tetramethylbenzidine (X-tra Kem-En-Tec, Taastrup, Denmark). The reaction was stopped with HCl (1 mol/L), and optical density was measured at 450 nm using a multilabel plate reader (Chameleon: Hidex, Turku, Finland). Assay characterization showed: 50% inhibition of signal was obtained at 10 pmol/L standard peptide; recovery of peptide added to human CSF was > 80%; intra- and inter-assay coefficients of variation (CV%) were 4 and 10%, respectively. Serial sample dilutions showed a profile parallel to the standard curve (deviation: < 10%). When data were tested vs. duration of sample storage (at − 80 °C, 1 to > 10 years), no correlation was revealed. To gain some insight as to the specificity of the assay for N-terminally cleaved peptides, versus the same sequence within N-terminally extended forms (possibly including the VGF precursor), a synthetic peptide containing an additional N-terminal Arg residue was tested in the assay (corresponding to Arg_372_ in the di-basic site: human VGF Arg_371_-Arg_372_ immediately preceding the natural peptide VGF_373–417_ and iplicated in its N-terminal cleavage). The data showed a < 0.5% cross-reactivity for this peptide, hence indicating a high specificity of the assay for the N-terminally cleaved peptide.

#### SRM analysis of VGF

For SRM analysis of VGF in CSF samples, 200 μL of CSF was spiked with TEAB buffer and a quantitative protein epitope signature tag (QPrEST, kindly provided by Atlas Antibodies AB, #QPrEST20926) of VGF as internal standard. Samples were reduced and alkylated with 1 mM TCEP and 1 mM CAA at 95 °C for 10 min. Proteins were digested for 16 h at 37 °C by adding 1.2 μg trypsin/LysC (Promega). Digestion was stopped by addition of 800 μL 1.25% TFA and peptides were transferred to strong cation exchange STAGE-Tips [32] by centrifugation. Peptides were washed with 0.2% TFA followed by 75 mM ammonium acetate/20% acetonitrile/0.5% formic acid and eluted with 125 mM ammonium acetate/20% acetonitrile/0.5% formic acid. After vacuum drying, peptides were dissolved in 30 μL 6% acetonitrile/0.1% TFA and analyzed by LC-SRM. Analysis of VGF was performed with a QTRAP6500 mass spectrometer (AB Sciex), Eksigent MicroLC200 and Agilent 1260 HPLC pump. Peptides were loaded on a C18 PepMap100, 5 μm, 0.3 × 5 mm trap column (Thermo Fisher Scientific). Separation was performed on an Eksigent HALO Fused-core C18, 2.7 μm, 0.5 × 100 mm column at 40 °C with mobile phase A: 4% DMSO/0.1% formic acid, and mobile phase B: 4% DMSO/96% acetonitrile/0.1% formic acid and a linear gradient from 1 to 30%B within 9.85 min. The following transitions of the proteotypic VGF peptide AQEEAEAEER (aa586–595) were measured: 581.3–962.4 (y8), 581.3–833.4 (y7), 581.3–704.3 (y6) (light peptide); 586.3–972.4 (y8), 586.3–843.4 (y7), 586.3–714.3 (y6) (heavy peptide). For relative quantification, the light-to-heavy (L/H) peptide ratio (mean of the three transitions) was calculated using Skyline v4.2. CSF QC samples were included in each run. Intra-assay CVs were 5.1–7.9%.

### Statistical analysis

All statistical analyses were performed in R v.3.5.1 ‘Feather Spray’. Demographics were compared using Student’s t-test, Wilcoxon signed-rank test or Fisher’s Exact Test. Correlations between identified CSF biomarkers and age, sex and MMSE were assessed with Spearman partial correlation, adjusted for cohort. For the validation of the identified candidate biomarker levels in related neurodegenerative diseases (cohort 3A), all protein levels were first normalized according to the mean and standard deviation values of their corresponding control group. The obtained z-scores were compared using general linear models corrected for age. In addition, we performed random forest analyses [33] with the R package randomForest using automated parameter optimization with the caret package to identify a subset of candidate markers that best distinguished DLB from all other groups. We used Monte Carlo sampling with replacement to sample test groups to generate a random forest classifier with the minimum number of predictors, and used the left out data to test the resulting classifier. This procedure was repeated for 1000 iterations. Diagnostic groups differed in sample size, and to avoid class imbalance effects on classifier performance, we down-sampled the larger group to the same size as the smaller group for training. Classification performance on the test data was determined with accuracy, sensitivity and specificity using the R package “caret”. In order to visualize separation in diagnoses for the combined top selected proteins, we performed k-means clustering on these proteins, including also age. Optimal number of clusters was determined on the within cluster sums of squares, based on the point after which only minimal additional variance was explained. Cluster solution was plotted against the first two dimensions. Finally, general linear models were performed to compare CSF levels of VGF and NPTX2 between DLB patients and controls in cohort 3B. A FDR-value < 0.05 was considered statistically significant.

## Results

### Patient characteristics

Table 1 displays the demographics and CSF characteristics of DLB patients and controls included in cohort 1, 2 & 3B. The diagnostic groups had similar age and sex distributions. DLB patients had lower MMSE cores compared to controls. Per inclusion criteria, all DLB patients in the cohort 1 and all controls had normal AD biomarker levels in CSF, whereas almost half of the DLB patients in cohort 2 & 3B had a CSF AD profile.

**Table 1:** Demographics and CSF characteristics of DLB patients and controls

	**Cohort 1 (*n*** **= 40)**		**Cohort 2 (*n*** **= 30)**		**Cohort 3B (*n*** **= 76)#**	
	**DLB (*n*** **= 20)**	**Controls (*n*** **= 20)**	**DLB (*n* = 17)**	**Controls (*n*** **= 13)**	**DLB (*n*** **= 48)**	**Controls (*n*** **= 28)**
**Age,** yr range [min-max]	65.3 ± 5.8 [54.1–76.5]	65.1 ± 5.4 [53.9–74.0]	66.9 ± 7.5 [53.9–74.0]	65.6 ± 8.5 [52.4–76.7]	67.8 ± 6.3* [54.1–78.4]	64.1 ± 5.8 [53.9–74.0]
**Male sex**	17 (85%)	17 (85%)	13 (76%)	9 (69%)	42 (88%)	24 (86%)
**Symptom duration,** yr	3 [2–4]	N/A	2 [2–4]	N/A	2 [1–4]	N/A
**MMSE**	23 [21–26]***	28 [27–29]	26 [21–28]*	29 [28–30]	23 [21–26]***	28 [27–30]
**CSF AD biomarkers**						
**Aß**_**1–42**_**,** (pg/ml) **Abnormal**	846 [637–1011] 0 (0%)	820 [691–1039] 0 (0%)	611 [478–942]* 8 (47%)	959 [932–1054] 0 (0%)	660 [536–871]** 13 (27%)	856 [691–1027] 0 (0%)
**total tau,** (pg/ml) **Abnormal**	238 [200–286] 0 (0%)	209 [167–266] 0 (0%)	317 [268–599]** 8 (47%)	226 [194–253] 0 (0%)	299 [224–370]*** 11 (23%)	190 [156–257] 0 (0%)
**p-tau,** (pg/ml) **Abnormal**	37 [29–47] 0 (0%)	42 [31–47] 0 (0%)	46 [41–71] 7 (41%)	41 [37–49] 0 (0%)	47 [34–61]* 18 (37%)	38 [28–46] 0 (0%)
**APOE ε4 carrier**	9 (45%)	8 (40%)	10 (71%)**	2 (17%)	25 (55%)	10 (38%)

Data are presented as mean ± SD for normally distributed continuous variables, as median [first quartile – third quartile] for non-normally distributed continuous variables or as n (%) for categorical variables

CSF cutoff values were set on Aß_1–42_ < 550 pg/ml, total tau > 375 pg/ml, p-tau > 52 pg/ml [17]

Differences between DLB patients and controls were assessed with Student’s t-test, Wilcoxon signed-rank test or Fisher’s Exact Test.* *p* < 0.05, ** *p* < 0.01, *** *p* < 0.001

#NPTX2 ELISA: *n* = 76 (DLB: *n* = 48, Controls: *n* = 28); VGF ELISA: *n* = 66 (DLB: *n* = 44, Controls: *n* = 22); VGF SRM: *n* = 65 (DLB: *n* = 44, Controls: *n* = 21)

Abbreviations: *Aß*_*1–42*_ ß-Amyloid 1–42, *AD* Alzheimer’s disease, *APOE* Apoliproprotein E, *CSF* Cerebrospinal fluid, *DLB* Dementia with Lewy bodies, *MMSE* Mini mental State examination (score range 0–30), *N/A* Not applicable, *p-tau* Tau phosphorylated at threonine 181

### Phase 1: CSF biomarker discovery

**Fig. 2:**
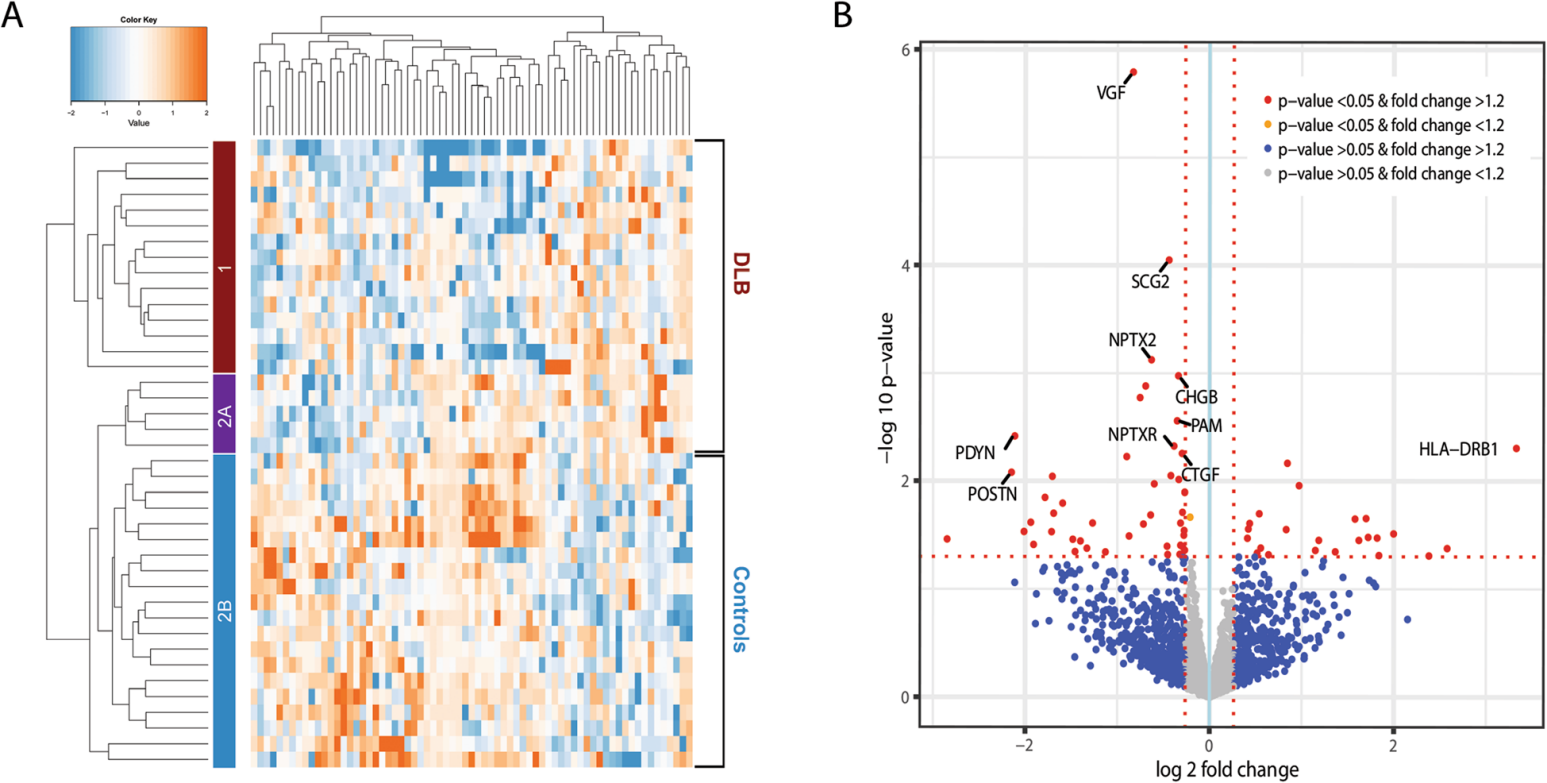
Results of discovery proteomics. **a** Heatmap and cluster analysis of differentially expressed proteins (*n* = 69) in cohort 1. The heatmap shows distinct patterns of up- and downregulated proteins in the clinical groups. The branching pattern of the dendrogram shows almost complete separation of patients with DLB from cognitively normal controls (35/40 (87.5%) were clustered correctly). Fifteen DLB patients were assigned to cluster 1 (red) and five DLB patients and 20 controls were assigned to cluster 2. The five DLB patients in cluster 2 clustered together in a small subgroup (cluster 2A, purple) and the controls clustered together in another subgroup (cluster 2B, blue). **b** Volcano plot representing the top biomarker candidates discriminating DLB from controls. The horizontal axis indicates log2 fold change. The vertical axis indicates − 10 log p-values. Each point represents a protein. Points at the far right- and left-hand sides of the plot have the largest fold changes, while those along the top of the plot are the most statistically significant. The non-axial red dotted vertical lines denote fold change thresholds of 1.2. The non-axial red dotted horizontal line denotes p-value threshold of 0.05. Proteins in red have a fold change > 1.2 and p-value < 0.05. The top-10 biomarker candidates are highlighted in the plot

In total, 1995 proteins were identified in the discovery cohort (cohort 1). A total of 69 unique proteins showed significantly different abundances (*p* < 0.05). Forty-six proteins were downregulated and 23 proteins were upregulated in DLB (Supplementary Table 1). Figure 2a shows the heatmap and cluster analysis of differentially expressed proteins. Hierarchical cluster analysis including the differentially expressed proteins revealed almost complete separate clustering of DLB patients and controls (87.5% were clustered correctly). The dendrogram illustrates the two distinct clusters: 15 DLB patients were assigned to cluster 1. Interestingly, the 5 DLB patients in cluster 2 clustered together in a subgroup (cluster 2A), while all 20 controls clustered together in subgroup 2B. The level of significance and the magnitude of changes of the quantitative data are visualized in a volcano plot (Fig. 2b).

### Phase 2: validation using identical proteomic workflow in an independent cohort

Next, we performed a replication in a completely independent second cohort (17 DLB patients, 13 controls) to validate the results (cohort 2, Table 1). Here, 1967 proteins were identified, of which 93 proteins were differentially expressed (*p* < 0.05, Supplementary Table 2). Overlap analysis between the differentially expressed proteins (*p* < 0.05) showed six proteins with same direction and magnitude of change in both cohorts, i.e. Neurosecretory protein (VGF), Secretogranin-2 (SCG2), Neuronal pentraxin-2 (NPTX2), Neuronal pentraxin receptor (NPTXR), Proenkephalin-B (PDYN) and ProSAAS (PCSK1N) (Table 2).

**Table 2:** Overlapping differentially expressed proteins between cohort 1 and 2

**Uniprot ID**	**Gene name**	**Protein name**	**Fold change in discovery cohort**	**Fold change in validation cohort**	**CSF Peer literature (see Suppl. Table** **3****for references)**
O15240	VGF	Neurosecretory protein VGF	−1.78	−1.41	↓ in AD, FTD and ALS ↑ in schizophrenia
P13521	SCG2	Secretogranin-2	−1.36	−1.30	↓ in MS ↓ in AD
P47972	NPTX2	Neuronal pentraxin-2	−1.55	−1.50	↓ in AD
O95502	NPTXR	Neuronal Pentraxin Receptor	−1.31	−1.32	↓ in AD
P01213	PDYN	Proenkephalin-B	−4.33	−8.78	↓ in AD
Q9UHG2	PCSK1N	ProSAAS	−1.22	−1.21	↓ in AD ↓ in FTD

Table lists the six CSF biomarker candidates for DLB. A positive fold change indicates that the protein is upregulated in the DLB group in contrast to the control group. A negative fold change indicates the protein is downregulated in the DLB group compared to the control group

Abbreviations: *AD* Alzheimer’s disease, *ALS* Amyotrophic lateral sclerosis, *DLB* Dementia with Lewy bodies, *FTD* Frontotemporal dementia, *MS* Multiple sclerosis, *NPTX2* Neuronal pentraxin 2, *NPTXR* Neuronal pentraxin receptor, PCSK1N, ProSAAS, *PDYN* Proenkephalin-B, *SCG2* Scretogranin-2, *VGF* Neurosecretory protein VGF

**Fig. 3:**
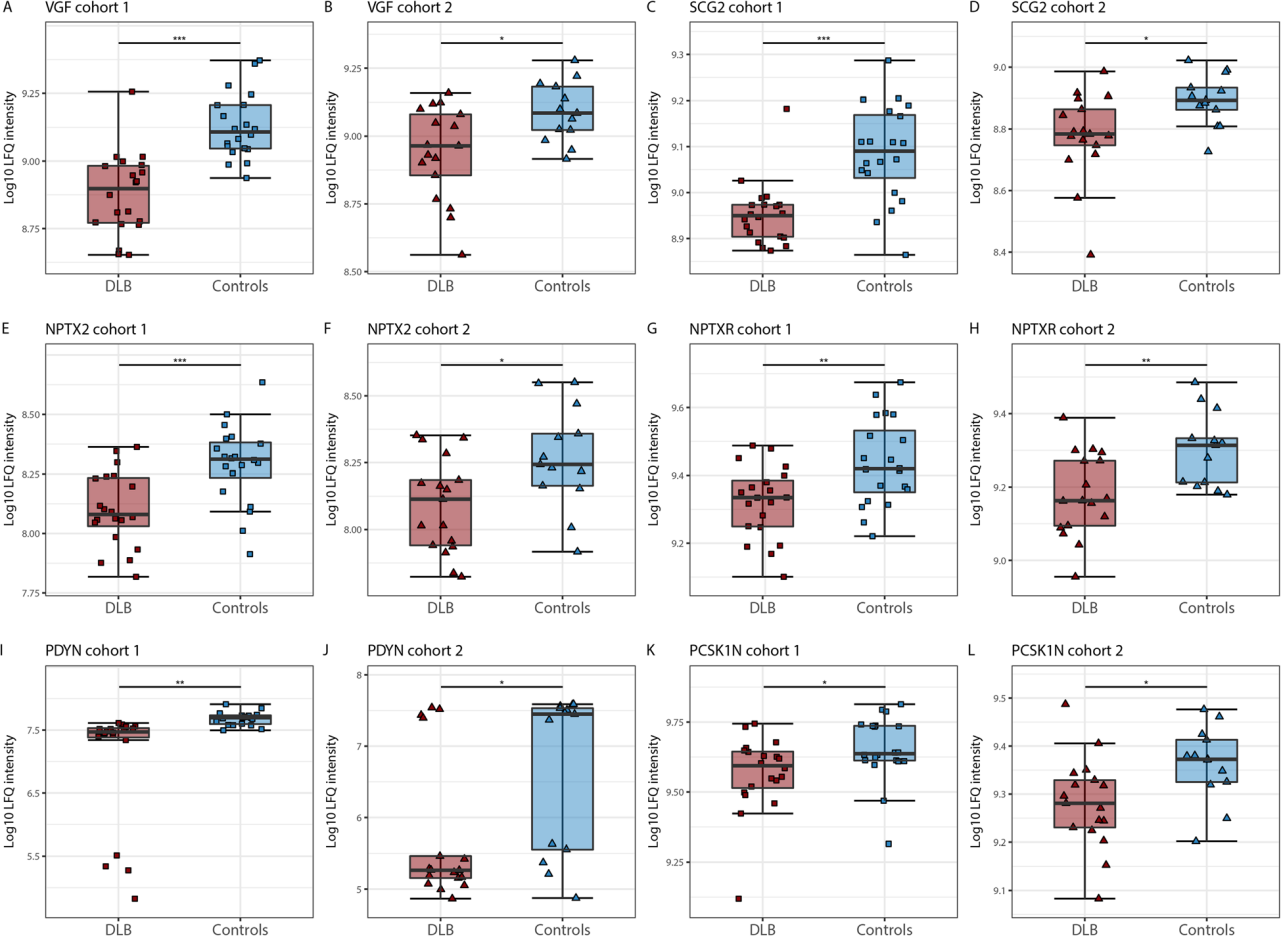
Box and Whisker plots of candidate CSF biomarkers for DLB. **a** Log 10 LFQ intensity of VGF in cohort 1, **b** VGF in cohort 2, **c**, **d** SCG, **e**, **f** NPTX2, **g**, **h** NPTXR, **i**, **j** PDYN, **k**, **l** PCSK1N. The line through the middle of the boxes corresponds to the median and the lower and the upper lines to the 25th and 75th percentile, respectively. The whiskers extend from the 5th percentile on the bottom to the 95th percentile on top. Differences between DLB patients and controls were assessed limma package available from the Bioconductor package, * *p* < 0.05, ** *p* < 0.01, *** *p* < 0.001. Abbreviations: DLB, Dementia with Lewy bodies; NPTX2, Neuronal pentraxin 2; NPTXR, Neuronal pentraxin receptor, PCSK1N, ProSAAS; PDYN, Proenkephalin-B; SCG2, Secretogranin-2; VGF, Neurosecretory protein

**Fig. 4:**
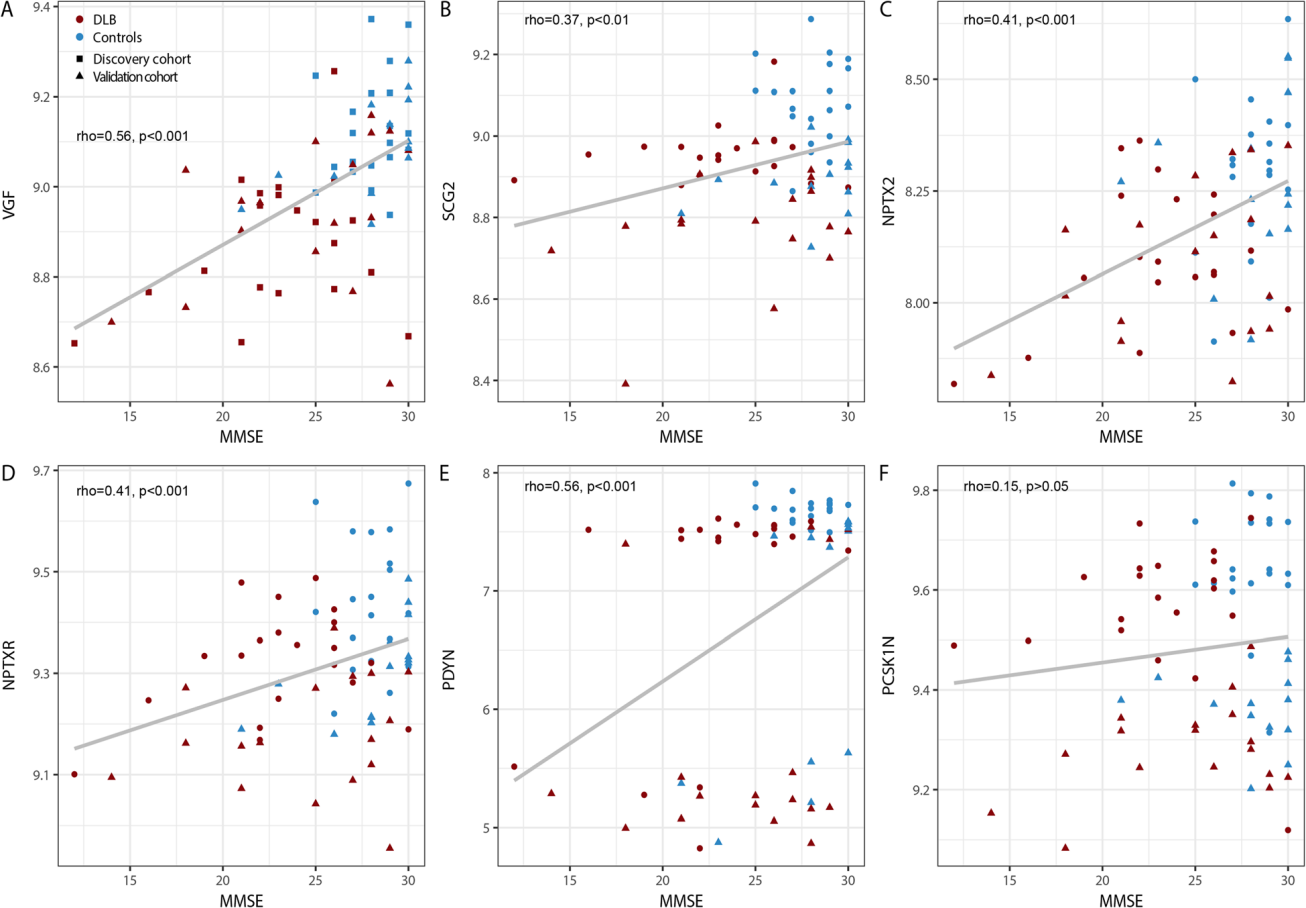
Associations between the six CSF candidate biomarkers for DLB and MMSE. Scatter plots of MMSE and CSF levels of (**a**) VGF (**b**) SCG2, (**c**) NPTX2, (**d**) NPTXR, (**e**) PDYN, (**f**) PCSK1N across DLB (red) and control groups (blue). Individual subject cohort 1 are depicted as squares and individual subjects from cohort 2 are depicted as triangles. Associations were assessed using Spearman partial correlation adjusted for cohort. To correct for multiple comparisons, p-values were corrected using a false discovery rate (FDR) correction. Abbreviations: DLB, Dementia with Lewy bodies; NPTX2, Neuronal pentraxin 2; NPTXR, Neuronal pentraxin receptor, PCSK1N, ProSAAS; PDYN, Proenkephalin-B; SCG2, Scretogranin-2; VGF, Neurosecretory protein VGF

Figure 3 shows the individual levels of these six candidate biomarkers in both cohort 1 and 2. CSF levels of all these proteins were lower in DLB patients compared to controls (all *p* < 0.05). Next, we explored whether these six candidate biomarkers were associated with age, sex and cognitive impairment. Partial spearman correlation analysis adjusted for cohort revealed that lower CSF levels of all proteins, except PCSK1N, were associated with lower MMSE scores at time of lumbar puncture (0.37 < r < 0.56, all *p* < 0.01; Fig. 4), whereas no associations were found with age and sex (data not shown).

### Phase 3A: validation of candidate CSF biomarkers in related neurodegenerative diseases

**Fig. 5:**
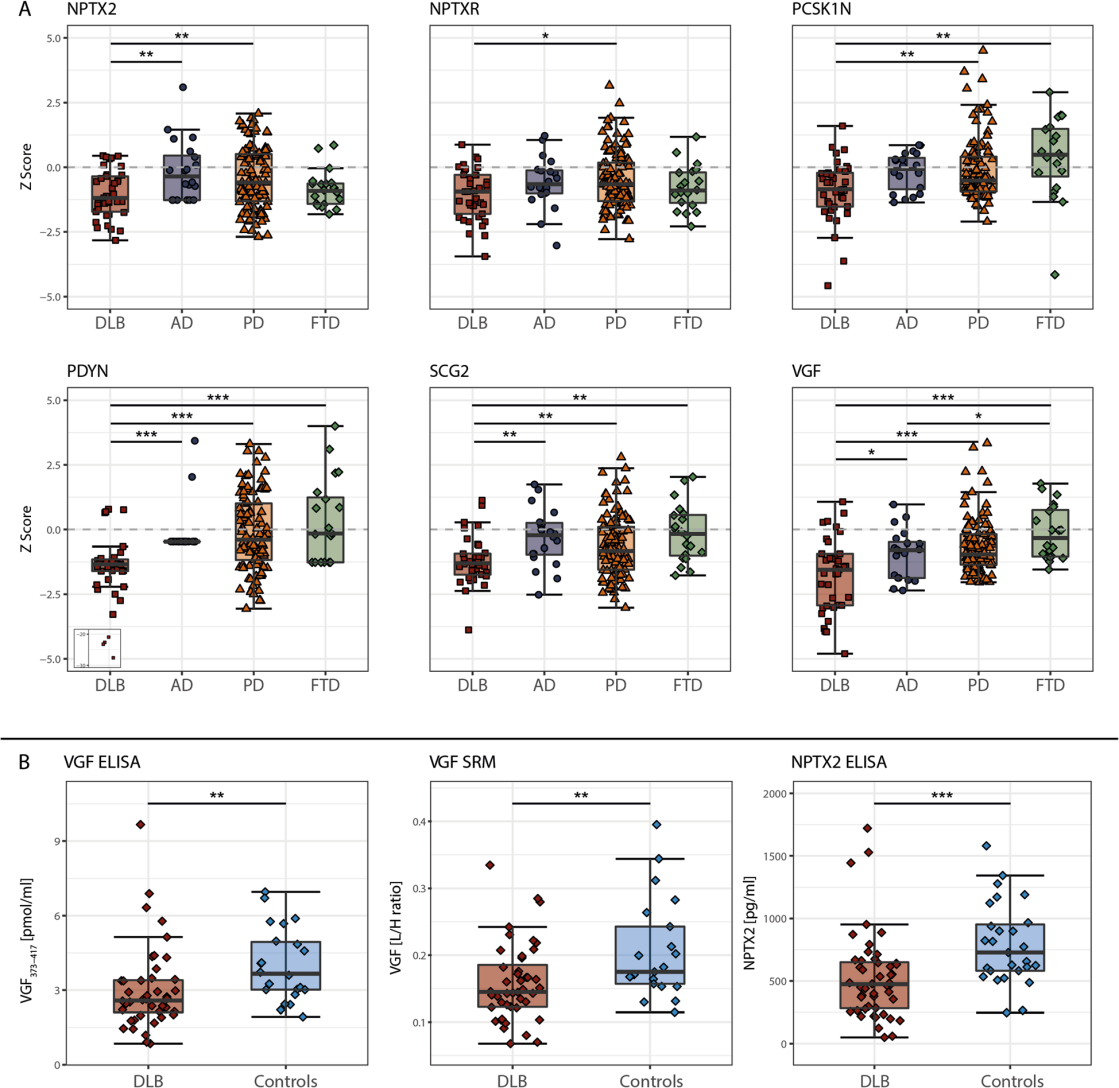
Validation of candidate biomarkers. **a** Differences in levels of candidate biomarkers between DLB and related neurodegenerative diseases. All protein levels were Z transformed according to the mean and standard deviation in controls, dotted line represents average protein levels for the control group. For PDYN, 4 outliers (z-score > 20) were illustrated in a box. Please note that the low variation in PDYN levels in AD patients is caused by lack of a measurable concentration.Differences were assessed with GLM corrected for age and a FDR correction was applied. * *p* < 0.05, ** *p* < 0.01, *** *p* < 0.001. **b** Validation of VGF and NPTX2 using orthogonal analytical methods. Levels of VGF_373–417_ (pmol/ml) were determined by ELISA, levels of VGF [LH/ratio] were determined with SRM and levels of NPTX2 (pg/ml) were determined with ELISA in CSF samples from DLB patients (*n* = 48) and controls (*n* = 28). The line through the middle of the boxes corresponds to the median and the lower and the upper lines to the 25th and 75th percentile, respectively. The whiskers extend from the 5th percentile on the bottom to the 95th percentile on top. Differences between DLB patients and controls were assessed with GLM. * *p* < 0.05, ** *p* < 0.01, *** *p* < 0.001. Abbreviations: AD, Alzheimer’s disease; DLB, Dementia with Lewy bodies; ELISA, enzyme-linked immunosorbent assays; FTD, frontotemporal dementia; NPTX2, Neuronal pentraxin 2; NPTXR, Neuronal pentraxin receptor, PCSK1N, ProSAAS; PD, Parkinson’s disease; PDYN, Proenkephalin-B; SCG2, Secretogranin-2; SRM, selected reaction monitoring; VGF, Neurosecretory protein VGF

Next, we investigated the candidate biomarker values in related neurodegenerative diseases, including AD, PD and FTD (Fig. 5a). CSF levels of the candidate biomarkers, except PCSK1N, were in general lower in all neurodegenerative patient groups compared to the control group. Protein levels were consistently lowest in DLB patients. Specifically, CSF NPTX2 levels were lower in DLB compared to both AD and PD (*p* < 0.05). CSF NPTXR levels were lower in DLB than in PD (*p* < 0.05). CSF levels of PCSK1N were lower in DLB compared to both PD and FTD (*p* < 0.05). CSF levels of PDYN, SCG2 and VGF were lower in DLB compared to all related neurodegenerative diseases studied (*p* < 0.05). CSF levels of all proteins were comparable between the other neurodegenerative disease, i.e. AD, PD and FTD, except VGF for which levels were lower in AD compared to FTD (*p* < 0.05). The identified markers still showed considerable overlap between groups, suggesting limited ability for diagnostic purposes as single markers. Therefore, we performed random forest analyses to study whether a combination of biomarkers improved discrimination between DLB and all non-DLB individuals. VGF, SCG2 and PDYN best differentiated between DLB and all non-DLB, with accuracy of 0.82, specificity of 0.83 and sensitivity of 0.69 (Table 3). To visualize separation in clinical diagnosis for the combined top selected proteins, we performed k-means clustering on these proteins, including also age (Supplementary Figure 1). Subsequently, we performed pairwise comparisons between DLB versus all other clinical groups using this model. Table 3 shows a summary of the pairwise diagnostic classification results. The panel discriminated DLB other clinical groups with accuracies ranging from 76 to 89%. The specificity of all pairwise comparisons was high (0.80–1.00) while sensitivity was moderate (0.72–0.85).

**Table 3:** Diagnostic performance of a biomarker panel for DLB versus other clinical groups

**DLB versus**	**Accuracy (95% CI)**	**Sensitivity (95% CI)**	**Specificity (95% CI)**
**All non-DLB**	0.82 (0.75–0.89)	0.69 (0.41–0.98)	0.83 (0.74–0.90)
**Controls**	0.84 (0.77–0.89)	0.77 (0.46–1.00)	0.85 (0.76–0.93)
**PD**	0.79 (0.69–0.87)	0.72 (0.43–0.93)	0.80 (0.66–0.90)
**AD**	0.89 (0.85–0.99)	0.85 (0.79–0.99)	1.00 (1.00–1.00)
**FTD**	0.76 (0.61–0.92)	0.73 (0.55–0.95)	0.86 (0.59–1.00)

All protein levels were Z transformed according to the mean and standard deviation in controls

Abbreviations: *AD* Alzheimer’s disease, *DLB* Dementia with Lewy bodies, *FTD* Frontotemporal dementia, *PD* Parkinson’s disease

### Phase 3B: validation of candidate CSF biomarkers by ELISA and SRM

Finally, VGF and NPTX2 were selected for validation in cohort 3 based on the availability of orthogonal analytical methods (ELISA and SRM). As shown in Fig. 5b, decreased levels of CSF VGF (VGF_373–417_ in ELISA) and NPTX2 were confirmed using these alternative analytical methods (*p* < 0.01 and *p* < 0.001, respectively).

## Discussion

Using a state-of-the-art rigorous proteomic approach and validation in a completely independent cohort, we identified and positively validated six promising CSF biomarker candidates for DLB, namely VGF, SCG2, NPTX2, NPTXR, PDYN and PCSK1N (proSAAS). All six biomarker candidates were downregulated in DLB and levels were consistently lowest in DLB patients compared to related neurodegenerative diseases studied, i.e. AD, PD and FTD. Additionally, we utilized machine learning to identify the biomarker panel best capable of classifying DLB patients. The combination of VGF, SCG2 and PDYN best differentiated between DLB and related neurodegenerative diseases with acceptable specificity and sensitivity. In a second validation step, we confirmed the decrease of CSF VGF (ELISA, SRM) and NPTX2 (ELISA) using orthogonal analytical techniques. Low CSF levels of all biomarker candidates, except PCSK1N, were associated with more pronounced cognitive decline. We will discuss these validated biomarker candidates below.

Three identified biomarker candidates (VGF, SCG2 and PCSK1N) are members of the chromogranin/secretogranin family and play a role in the regulated secretory pathway of peptides, hormones, neurotransmitters and growth factors. VGF topped the list of potential biomarker candidates. Biologically active peptides derived from VGF play an important role in diverse processes, for example, hormone, neurotrophin and neurotransmitter release, energy homeostasis and regulation of gastrointestinal function [34, 35]. Although VGF peptides have so far not been associated with DLB, previous proteomic studies observed changes in VGF peptides in the CSF of patients affected with several neurodegenerative and psychiatric disorders. In line with our findings in DLB, multiple VGF peptides were shown to be decreased in CSF from patients with AD, FTD (VGF_26–62_) or amyotrophic lateral sclerosis (ALS) (VGF_398–411_). In addition, VGF peptides were also reduced in brain tissue of patients with PD [36]. Conversely, CSF levels of VGF_23–62_ were increased in schizophrenia patients (see Supplementary Table 3 for an overview of the literature). The second biomarker candidate SCG2 is involved in the packing or sorting of peptide hormones and neuropeptides into secretory vesicles, plays a role in inflammatory responses and in the regulation of the blood pressure [34]. Consistent with our findings, reduced levels of CSF SCG2 in AD and multiple sclerosis (MS) have been reported (Supplementary Table 3). In view of the decrease of VGF and SCG2 in CSF of patients with different neurodegenerative disorders and their localization in synaptic vesicles, we propose that VGF and SCG2 are markers for synaptic degeneration. Third, PCSK1N, an inhibitor of prohormone convertase (PC) activity [34], has also been proposed as a CSF biomarker candidate for several neurological disorders. For example, reduced levels of CSF PCSK1N in AD and FTD have been reported (Supplementary Table 3). Moreover, several lines of evidence have implicated that PCSK1N blocks aggregation of Aβ_1–42_ and α-synuclein [37, 38], supporting a function of PCSK1N as a neuronal secretory chaperone in DLB.

NPTX2 and NPTXR are members of the neuronal pentraxin family [39]. The neuronal pentraxin family has not previously been related to DLB. However, NPTX2 and NPTXR were also reduced in AD (Supplementary Table 3). NPTX2 promotes formation of new excitatory synapses and regulation of AMPA-type receptors clustering at established synapses [40]. The altered levels of NPTX2 and NPTXR further substantiate the importance of synaptic dysfunction in the pathophysiology of DLB. In contrast to the results in AD and DLB, both the gene and tissue expression of NPTX2 were upregulated in PD [41]. We showed that CSF NPTX2 levels in DLB were lower compared to both AD and PD patients. The reduction of NPTX2 that is correlated with cognitive decline implicates a pathophysiological mechanism – failure of the adaptive function of pyramidal neurons to modify excitatory drive of fast spiking parvalbumin (PV) interneurons- that could potentially be targeted for therapeutics [42].

The sixth biomarker candidate that we identified and validated was PDYN. The large decrease (fold change of > 4) suggest that PDYN is an on/off marker (i.e. subjects either have low PDYN levels or have high PDYN levels). More DLB patients than controls have very low PDYN levels resulting in an average decreased expression in DLB (Fig. 3). PDYN is a precursor protein that is processed by PC1, PC2 and carboxypeptidase E to form different opioid neuropeptides (collectively referred to as dynorphins) [43]. The effects of dynorphins are mediated through two kinds of receptors: (1) κ-opioid receptor (KOP) and (2) NMDA or AMPA receptors. Dysregulation of the dynorphin/KOP system may contribute to behavioral abnormalities that are commonly shared by psychiatric disorders (i.e. decreased motivation and negative affect) [44], while non-opioid effects of dynorphins on NMDA or AMPA receptors could result in apoptosis and neurodegeneration [45]. Consistent with the decreased levels of PDYN in DLB in our study, reduced dynorphin levels have also been observed in CSF from AD patients (Supplementary Table 3) and in the amygdala of patients diagnosed with major depression and bipolar disorder [46]. Interestingly, a substantial loss of hypothalamic cells producing hypocretin, PDYN and NPTX2 has been found in patients with narcolepsy [47, 48]. Patients with narcolepsy suffer from symptoms that are also present in DLB, such as excessive daytime sleepiness, hypnagogic hallucinations and RBD [49]. The overlap in symptoms might suggests a common etiology between DLB and narcolepsy and indicates that PDYN and NPTX2 reduction may be important biological substrates underlying sleep-related symptoms in DLB.

The identification of these candidate biomarkers highlight the importance of synaptic dysfunction in DLB. This is in line with previous research indicating that this biological process is a central feature in DLB pathogenesis [50–52]. However, the mechanisms leading to synaptic dysfunction in DLB remain elusive. Growing evidence indicates that accumulation of α-synuclein at presynaptic sides may contribute towards explaining synaptic dysfunction in DLB [50, 51, 53, 54]. A prevailing hypothesis is that excessive α-synuclein leads to deficits in vesicular transport/trafficking resulting in functional impairment of neurotransmitter release at the synapse [50, 51, 53, 54]. Synaptic dysfunction is thought to precede neuronal degeneration in DLB, and may correlate more directly with cognitive decline than pathological hallmarks such as Lewy bodies [55]. Indeed, most of the identified biomarker candidates were associated with cognitive decline in DLB. Our results provide support to the link between cognitive performance and synaptic protein loss in DLB.

Loss of synapses and a decrease in synaptic proteins are constant features of neurodegenerative diseases. This is also supported by similar synaptic protein changes in post-mortem brain tissue revealed by proteomic analysis from patients with AD, PD dementia and DLB [55]. The identified candidate biomarkers may therefore not be selectively reduced in DLB. Although synaptic dysfunction and loss is evident in neurodegenerative diseases, our findings tentatively suggest that synaptic dysfunction appears to be more pronounced in DLB than in related neurodegenerative diseases, i.e. AD, PD and FTD, or may reflect differing synaptic deficits among neurodegenerative diseases. Of note, the downregulation of the identified proteins in DLB is unlikely to be caused by concomitant AD pathology, since all DLB patients in cohort 1 and most DLB patients in cohort 2B and 3B had normal CSF AD biomarker levels. Most candidate biomarkers, however, showed considerable overlap between diagnostic groups, suggesting limited ability for diagnostic purposes as single markers. Random forest analyses suggested that VGF, SCG2 and PDN combined could best differentiate between DLB and all non-DLB individuals with a high specificity and moderate sensitivity. Despite the somewhat lower sensitivity, a vital characteristic for a biomarker (panel) is its specificity, i.e. the ability of a biomarker (panel) to correctly identify all people who not have the condition of interest, in determining disease state. Clinical symptoms are quite sensitive, but lack specificity in terms of distinguishing DLB from other types of neurodegenerative diseases, therefore, the identified biomarker panel could importantly add to the clinical work-up of DLB. Possibly, other combinations of proteins measured with modern discovery studies may further aid in differentiating between diagnoses, and future research in larger sample sizes should further investigate this question.

Among the strengths of the current study are the use of a rigorous in-depth proteomic approach, replication in an independent cohort, validation of the biomarker candidates in related neurodegenerative diseases, including AD, PD and FTD, validation of a subset of biomarker candidates using orthogonal techniques, and the strict inclusion criteria for patients and controls. For example, an abnormal DAT-SPECT scan and normal AD biomarker levels were obligatory for all DLB patients in the discovery cohort. On the other hand, DLB patients in the validation cohorts were more heterogeneous, i.e. DLB patients were more representative of DLB patients in daily memory clinic practice, as they were not selected based on their CSF AD biomarker values and almost half of the DLB patients had a CSF profile compatible with AD (in line with previous literature [56]). The use of this study design increases the generalizability of our findings. These strengths make the current study the most comprehensive proteomic analysis in DLB so far. Our study has nonetheless also limitations. A potentially important drawback is that the proteomic pipeline is biased towards the identification of more abundant proteins. This is particularly a problem in mass spectrometry-based proteomic analysis of CSF, since most proteins secreted from the brain into the CSF (e.g. cytokines and neuropeptides) have low concentrations (~ 150 μg/mL). For example, several known key pathological determinants of DLB, including α-synuclein and Aβ_1–42_, were not detected, since their concentration are below the typical limit of detection of mass spectrometry methods. In addition, these proteins can be highly post-translationally modified, which further compromises mass spectrometry-based identification by default search strategies. Hence, the possibility cannot be excluded that we may have missed some potentially interesting proteins. Furthermore, proteomic data of the PD patients were obtained from a different mass spectrometry platform than proteomic data of the AD, DLB and FTD patients, which could have introduced some noise. However, normalization of the biomarker candidate values relative to the corresponding control group could restrict the methodological differences. Additionally, the random forest classifier was generated and tested in the same cohort which may lead to over-optimistic classification results. Although we have used bootstrapping to reduce such over-fitting, larger cohorts are needed to validated these findings as well as to examine the added diagnostic value of the (combination of) candidate proteomic markers in relation to the established AD biomarkers and α-synuclein. Such a study would be of tremendous value to the field by optimizing biomarker panel for fit-for-use purposes, as well as to evaluate the role of synaptic dysfunction in the pathogenesis of neurodegeneration.

## Conclusion

In conclusion, we identified and positively validated six novel proteins (VGF, SCG2, NPTX2, NPTXR, PDYN and PCSK1N) as promising biomarkers for DLB. Our results might suggest that these candidate biomarkers, particularly when used as a panel, show promise to improve diagnostic accuracy for DLB, which should be explored in future prospective validation studies. Moreover, our validation using orthogonal techniques (i.e. high-throughput immunoassays or SRM) of a subset of the candidate biomarkers revealed by the proteomics approach supports the robustness of our findings. Therefore, future studies should include a replication in independent cohorts including patients with different neurodegenerative diseases (at least: DLB, AD, PD), and testing our panel of synaptic biomarkers, in combination with the AD biomarkers, using such higher throughput techniques (e.g. SRM). Identification of these candidate biomarkers also strengthens the importance of synaptic dysfunction in the pathophysiology of DLB, which warrant further research as potential therapeutic target. On the applicative and clinical side, the identification of novel CSF biomarkers for DLB, can be expected to enhance clinical diagnostic accuracy, especially early in the disease course, and might thereby accelerate the development of new disease-modifying and neuroprotective agents.

## Supplementary information

**Additional file 1.**

## Data Availability

The datasets used and/or analyzed during the current study are available from the corresponding author on reasonable request.

## Abbreviations

Aβ_1–42_: Amyloid-β 1–42
AD: Alzheimer’s disease
ALS: Amyotrophic lateral sclerosis
CSF: Cerebrospinal fluid
DaT-SPECT: ^123^I[FP-CIT] single photon emission computed tomography
DLB: Dementia with Lewy bodies
ELISA: Enzyme-linked immunosorbent assays
FTD: Frontotemporal dementia
MMSE: Mini-Mental State Examination
MS: Multiple sclerosis
NPTX2: Neuronal pentraxin 2
NPTXR: Neuronal pentraxin receptor
PCSK1N: ProSAAS
PD: Parkinson’s disease
PDYN: Proenkephalin-B
p-tau: Tau phosphorylated at threonine 181
RBD: REM sleep behavior disorder
REM: Rapid eye movement
SCG2: Secretogranin-2
SRM: Selected reaction monitoring
t-tau: Total tau
VGF: Neurosecretory protein VGF
