# Supplementary material for: Identification of novel cerebrospinal fluid biomarker candidates for dementia with Lewy bodies: a proteomic approach
Source: Mol Neurodegener. 2020 Jun 18;15:36. doi: 10.1186/s13024-020-00388-2 (PMC7301448; doi:10.1186/s13024-020-00388-2)
Supplement: Supplementary file 1 — Additional file 1. [file 13024_2020_388_MOESM1_ESM.docx]

# Additional File

**Identification of novel cerebrospinal fluid biomarker candidates for dementia with Lewy bodies: a proteomic approach**

Inger van Steenoven^1*^, Marleen J.A. Koel-Simmelink^2*^, Leonie J.M. Vergouw^3^, Betty Tijms^1^, Sander R. Piersma^4^, Thang V. Pham^4^, Claire Bridel^2^, Gian-Luca Ferri^5^, Cristina Cocco^5^, Barbara Noli^5^, Paul F. Worley^6,7^, Mei-Fang Xiao^6^, Desheng Xu^6^, Patrick Oeckl^8^, Markus Otto^8^, Wiesje M. van der Flier^1,9^, Frank Jan de Jong^3^, Connie R. Jimenez^4^, Afina W. Lemstra^1^, and Charlotte E. Teunissen^2^.

^1^ Alzheimer Center Amsterdam, Department of Neurology, Amsterdam Neuroscience, Vrije Universiteit Amsterdam, Amsterdam UMC, Amsterdam, The Netherlands

^2^ Neurochemistry Laboratory and Biobank, Department of Clinical Chemistry, Amsterdam Neuroscience, Vrije Universiteit Amsterdam, Amsterdam UMC, Amsterdam, The Netherlands

^3^ Alzheimer Center Erasmus MC, Department of Neurology, Erasmus Medical Center, Rotterdam, The Netherlands

^4^ OncoProteomics Laboratory, Department of Medical Oncology, Vrije Universiteit Amsterdam, Amsterdam UMC, Amsterdam, The Netherlands

^5^ NEF-laboratory, Department of Biomedical Sciences, University of Cagliari, Monserrato, Italy

^6^ Solomon H. Snyder Department of Neuroscience, Johns Hopkins University School of Medicine, Baltimore, United States

^7^ Department of Neurology, Johns Hopkins University School of Medicine, Baltimore, United States

^8^ Department of Neurology, Ulm University Hospital, Ulm, Germany

^9^ Department of Epidemiology and Biostatistics, Amsterdam Neuroscience, Vrije Universiteit Amsterdam, Amsterdam UMC, Amsterdam, The Netherlands

**Supplementary Table 1.** Differentially expressed proteins in cohort 1 based on mass spectrometry data analysis

| **Uniprot Accession** | **Gene name** | **Protein name** | **Sequence coverage (%)** | **Fold change** | **p-value** | **q-value** | **# DLB samples** | **# Control samples** |
| --- | --- | --- | --- | --- | --- | --- | --- | --- |
| **O15240** | **VGF** | **Neurosecretory protein VGF** | **63.4** | **-1.777** | **1.62E-06** | **0.003** | **20** | **20** |
| **P13521** | **SCG2** | **Secretogranin-2** | **52.2** | **-1.357** | **8.96E-05** | **0.089** | **20** | **20** |
| **P47972** | **NPTX2** | **Neuronal pentraxin-2** | **43.9** | **-1.548** | **0.001** | **0.498** | **20** | **20** |
| **P05060** | **CHGB** | **Secretogranin-1** | **71.5** | **-1.265** | **0.001** | **0.524** | **20** | **20** |
| CON__ENSEMBL:ENSBTAP00000006074 | | | 16.7 | -1.618 | 0.001 | 0,524 | 0 | 1 |
| P60842 | EIF4A | Eukaryotic initiation factor 4A | 20 | 0,089 | 0.002 | 0.559 | 0 | 1 |
| **P19021;** | **PAM** | **Peptidyl-glycine alpha-amidating monooxygenase** | **56.2** | 0,498 | **0.003** | **0.789** | **20** | **20** |
| **P01213** | **PDYN** | **Proenkephalin-B** | **42.9** | 0,524 | **0.004** | **0.954** | **16** | **20** |
| **O95502** | **NPTXR** | **Neuronal pentraxin receptor** | **67** | **-1.305** | **0.005** | **0.984** | **20** | **20** |
| **P13760** | **HLA-DRB1** | **HLA class II histocompatibility antigen. DRB1-4 beta chain** | **44** | **10.047** | **0.005** | **0.984** | **14** | **5** |
| **P29279-** | **CTGF** | **Connective tissue growth factor** | **31.7** | **-1.229** | **0.006** | **0.985** | **20** | **20** |
| P04440 | HLA-DPB1 | HLA class II histocompatibility antigen. DP beta 1 chain | 29.1 | -1.864 | 0.006 | 0.985 | 0 | 1 |
| Q9UKZ9 | PCOLCE2 | Procollagen C-endopeptidase enhancer 2 | 6.5 | 1.795 | 0.007 | 0.985 | 1 | 0 |
| **Q15063** | **POSTN** | **Periostin** | **45.8** | **-4.435** | **0.008** | **0.985** | **10** | **17** |
| **P49747** | **COMP** | **Cartilage oligomeric matrix protein** | **53.8** | **-1.337** | **0.009** | **0.985** | **20** | **20** |
| P55001 | MFAP2 | Microfibrillar-associated protein 2 | 12.4 | -3.262 | 0.009 | 0.985 | 0 | 5 |
| **Q9BXJ3** | **C1QTNF4** | **Complement C1q tumor necrosis factor-related protein 4** | **57.8** | **-1.260** | **0.010** | **0.985** | **20** | **20** |
| Q9BQ51 | PDCD1LG2 | Programmed cell death 1 ligand 2 | 12.6 | -1.516 | 0.011 | 0.985 | 0 | 1 |
| Q504Y2 | PKDCC | Extracellular tyrosine-protein kinase PKDCC | 17.4 | 1.959 | 0.011 | 0.985 | 2 | 0 |
| **Q8WXD2** | **SCG3** | **Secretogranin-3** | **74.4** | **-1.204** | **0.013** | **0.985** | **20** | **20** |
| **Q5VSG8** | **MANEAL** | **Glycoprotein endo-alpha-1.2-mannosidase-like protein** | **51.4** | **-3.448** | **0.014** | **0.985** | **15** | **20** |
| A6NL88 | SHISA7 | Protein shisa-7 | 7.4 | -3.020 | 0.016 | 0.985 | 16 | 20 |
| **P02452** | **COL1A1** | **Collagen alpha-1(I) chain** | **26.1** | **-1.226** | **0.019** | **0.985** | **20** | **20** |
| Q9ULH4 | LRFN2 | Leucine-rich repeat and fibronectin type-III domain-containing protein 2 | 16 | -3.229 | 0.020 | 0.985 | 2 | 9 |
| P30456 | HLA-A | HLA class I histocompatibility antigen. A-43 alpha chain | 54 | 1.453 | 0.020 | 0.985 | 1 | 0 |
| Q68DQ2 | CRYBG3 | Very large A-kinase anchor protein | 1.6 | -1.557 | 0.021 | 0.985 | 0 | 1 |
| Q16610 | ECM1 | Extracellular matrix protein 1 | 75.7 | -1.158 | 0.022 | 0.985 | 20 | 20 |
| **P15509** | **CSF2RA** | **Granulocyte-macrophage colony-stimulating factor receptor subunit alpha** | **36.9** | **3.247** | **0.022** | **0.985** | **19** | **13** |
| P06703 | S100A6 | Protein S100-A6 | 16.7 | 2.985 | 0.022 | 0.985 | 4 | 0 |
| Q6PCB0 | VWA1 | von Willebrand factor A domain-containing protein 1 | 41.1 | -3.837 | 0.024 | 0.985 | 4 | 10 |
| **P04275;** | **VWF** | **von Willebrand factor;von Willebrand antigen 2** | **48.2** | **-1.244** | **0.024** | **0.985** | **20** | **20** |
| Q16849 | PTPRN | Receptor-type tyrosine-protein phosphatase-like N | 19.6 | -2.408 | 0.024 | 0.985 | 18 | 20 |
| Q6JBY9 | RCSD1 | CapZ-interacting protein | 9.4 | 1.352 | 0.025 | 0.985 | 0 | 1 |
| Q4ZIN3 | TMEM259 | Membralin | 3.4 | -1.644 | 0.025 | 0.985 | 0 | 1 |
| **P01031** | **C5** | **Complement C5** | **63.4** | **1.339** | **0.028** | **0.985** | **20** | **20** |
| P05783 | KRT18 | Keratin, type I cytoskeletal 18 | 24.9 | 1.781 | 0.028 | 0.985 | 1 | 0 |

**Supplementary Table 1.** Differentially expressed proteins in cohort 1 based on mass spectrometry data analysis (continued)

| **Uniprot Accession** | **Gene name** | **Protein name** | **Sequence coverage (%)** | **Fold change** | **p-value** | **q-value** | **# DLB samples** | **# Control samples** |
| --- | --- | --- | --- | --- | --- | --- | --- | --- |
| **Q15768** | **EFNB3** | **Ephrin-B3** | **36.5** | **-1.211** | **0.028** | **0.985** | **20** | **20** |
| **P01859** | **IGHG2** | **Ig gamma-2 chain C region** | **36.2** | **-4.036** | **0.029** | **0.985** | **8** | **15** |
| **Q14DG7** | **TMEM132B** | **Transmembrane protein 132B** | **20.2** | **-3.279** | **0.029** | **0.985** | **13** | **18** |
| **P61812** | **TGFB2** | **Transforming growth factor beta-2** | **27.8** | **3.995** | **0.031** | **0.985** | **13** | **7** |
| **P08123** | **COL1A2** | **Collagen alpha-2(I) chain** | **25.5** | **-1.213** | **0.032** | **0.985** | **20** | **20** |
| **P16519** | **PCSK2** | **Neuroendocrine convertase 2** | **28.9** | **-1.833** | **0.032** | **0.985** | **19** | **20** |
| Q9NY56 | OBP2A | Odorant-binding protein 2a | 35.9 | 3.300 | 0.033 | 0.985 | 4 | 0 |
| **Q06141** | **REG3A** | **Regenerating islet-derived protein 3-alpha** | **41.7** | **3.528** | **0.033** | **0.985** | **18** | **12** |
| **Q9Y2E5** | **MAN2B2** | **Epididymis-specific alpha-mannosidase** | **42.8** | **1.330** | **0.034** | **0.985** | **20** | **20** |
| **P07478** | **PRSS2** | **Trypsin-2** | **12.1** | **-7.192** | **0.034** | **0.985** | **13** | **18** |
| **Q9NY33** | **DPP3** | **Dipeptidyl peptidase 3** | **40.8** | **-2.795** | **0.035** | **0.985** | **14** | **18** |
| **Q63HQ2** | **EGFLAM** | **Pikachurin** | **38.4** | **3.074** | **0.035** | **0.985** | **20** | **17** |
| P55087 | AQP4 | Aquaporin-4 | 15.3 | 2.274 | 0.035 | 0.985 | 4 | 0 |
| **Q15493** | **RGN** | **Regucalcin** | **53.2** | **-2.640** | **0.036** | **0.985** | **15** | **20** |
| **P52565** | **ARHGDIA** | **Rho GDP-dissociation inhibitor 1** | **22.5** | **-3.752** | **0.039** | **0.985** | **5** | **11** |
| **Q9UBX7** | **KLK11** | **Kallikrein-11** | **47.6** | **-1.244** | **0.039** | **0.985** | **20** | **20** |
| **Q9NTU7** | **CBLN4** | **Cerebellin-4** | **37.3** | **-1.377** | **0.040** | **0.985** | **20** | **20** |
| **Q9UHG2** | **PCSK1N** | **ProSAAS** | **68.5** | **-1.224** | **0.040** | **0.985** | **20** | **20** |
| Q8IXA5 | SPACA3 | Sperm acrosome membrane-associated protein 3 | 7.5 | 1.471 | 0.042 | 0.985 | 1 | 0 |
| P62837 | UBE2D2 | Ubiquitin-conjugating enzyme E2 D2 | 14.3 | 5.977 | 0.042 | 0.985 | 11 | 5 |
| **Q9BUD6** | **SPON2** | **Spondin-2** | **36.3** | **-2.514** | **0.042** | **0.985** | **17** | **20** |
| **O14793** | **MSTN** | **Growth/differentiation factor 8** | **44.5** | **-1.205** | **0.044** | **0.985** | **20** | **20** |
| Q9NS68 | TNFRSF19 | Tumor necrosis factor receptor superfamily member 19 | 8.9 | 2.217 | 0.044 | 0.985 | 4 | 1 |
| **Q9Y6C2** | **EMILIN1** | **EMILIN-1** | **20.4** | **2.575** | **0.045** | **0.985** | **17** | **12** |
| Q4LDE5 | SVEP1 | Sushi. von Willebrand factor type A. EGF and pentraxin domain-containing protein 1 | 9.5 | -2.750 | 0.045 | 0.985 | 8 | 14 |
| **Q6UY11** | **DLK2** | **Protein delta homolog 2** | **22.2** | **-2.187** | **0.045** | **0.985** | **17** | **20** |
| Q30134 | HLA-DRB1 | HLA class II histocompatibility antigen. DRB1-8 beta chain | 33.5 | 1.430 | 0.046 | 0.985 | 1 | 0 |
| **Q9UKM7** | **MAN1B1** | **Endoplasmic reticulum mannosyl-oligosaccharide 1.2-alpha-mannosidase** | **33.8** | **-1.250** | **0.048** | **0.985** | **20** | **20** |
| **Q92932** | **PTPRN2** | **Receptor-type tyrosine-protein phosphatase N2** | **21.5** | **-1.230** | **0.048** | **0.985** | **20** | **20** |
| O60486 | PLXNC1 | Plexin-C1 | 7.1 | -1.369 | 0.048 | 0.985 | 0 | 1 |
| P35613 | BSG | Basigin | 39.2 | 1.558 | 0.048 | 0.985 | 1 | 0 |
| **O94856** | **NFAS** | **Neurofascin** | **51.1** | **3.576** | **0.049** | **0.985** | **14** | **9** |
| **P02788** | **LTF** | **Lactotransferrin** | **66.5** | **5.211** | **0.049** | **0.985** | **18** | **13** |

*List of 69 differentially expressed proteins in CSF from patients with DLB compared to CSF from cognitively normal controls (p<0.05)*

*Proteins in bold (n=44) also fulfilled the predefined criteria for candidate biomarkers (p<0.05, fold change >1.2, >20% sequence coverage, detected in at least 50% of DLB patients or controls).*

**Supplementary Table 2.** Differentially expressed proteins in cohort 2 based on mass spectrometry data analysis

| **Uniprot Accession** | **Gene name** | **Protein name** | **Sequence coverage (%)** | **Fold change** | **p-value** | **q-value** | **# DLB samples** | **# control samples** |
| --- | --- | --- | --- | --- | --- | --- | --- | --- |
| **P05413** | **FABP3** | **Fatty acid-binding protein, heart** | **75.9** | **1.494** | **0.001** | **0.895** | **17** | **13** |
| **P09936** | **UCHL1** | **Ubiquitin carboxyl-terminal hydrolase isozyme L1** | **60.1** | **16.897** | **0.001** | **0.895** | **15** | **5** |
| **P13489** | **RNH1** | **Ribonuclease inhibitor** | **30.4** | **7.337** | **0.002** | **0.895** | **11** | **2** |
| **P63104** | **YWHAZ** | **14-3-3 protein zeta/delta** | **62.9** | **1.422** | **0.003** | **0.895** | **17** | **13** |
| **P62258** | **YWHAE** | **14-3-3 protein epsilon** | **79.2** | **1.309** | **0.003** | **0.895** | **17** | **13** |
| **P18669** | **PGAM1** | **Phosphoglycerate mutase 1** | **57.1** | **1.212** | **0.004** | **0.895** | **17** | **13** |
| **P52565** | **ARHGDIA** | **Rho GDP-dissociation inhibitor 1** | **22.5** | **4.740** | **0.005** | **0.895** | **17** | **8** |
| **P35080** | **PFN2** | **Profilin-2** | **37.9** | **1.253** | **0.005** | **0.895** | **17** | **13** |
| **P31946** | **YWHAB** | **14-3-3 protein beta/alpha** | **53.7** | **1.276** | **0.005** | **0.895** | **17** | **13** |
| **O43396** | **TXNL1** | **Thioredoxin-like protein 1** | **23.5** | **6.869** | **0.005** | **0.895** | **12** | **3** |
| **P00338** | **LDHA** | **L-lactate dehydrogenase A chain** | **62** | **1.297** | **0.006** | **0.895** | **17** | **13** |
| **Q9H008** | **LHPP** | **Phospholysine phosphohistidine inorganic pyrophosphate phosphatase** | **34.8** | **4.668** | **0.007** | **0.895** | **17** | **9** |
| **O95502** | **NPTXR** | **Neuronal pentraxin receptor** | **67** | **-1.321** | **0.008** | **0.895** | **17** | **13** |
| **Q9Y6R7** | **FCGBP** | **IgGFc-binding protein** | **50.6** | **1.523** | **0.008** | **0.895** | **17** | **13** |
| **Q9H7C9** | **AAMDC** | **Mth938 domain-containing protein** | **53.3** | **6.141** | **0.010** | **0.895** | **9** | **1** |
| **Q9H4F8** | **SMOC1** | **SPARC-related modular calcium-binding protein 1** | **35.3** | **1.406** | **0.010** | **0.895** | **17** | **13** |
| Q99969 | RARRES2 | Retinoic acid receptor responder protein 2 | 74.2 | 1.189 | 0.011 | 0.895 | 17 | 13 |
| Q13591 | SEMA5A | Semaphorin-5A | 7.9 | -1.715 | 0.011 | 0.895 | 0 | 1 |
| **O15240** | **VGF** | **Neurosecretory protein VGF** | **63.4** | **-1.413** | **0.012** | **0.895** | **17** | **13** |
| P09493 | TPM1 | Tropomyosin alpha-1 chain | 25.7 | 1.726 | 0.012 | 0.895 | 1 | 0 |
| **P61981** | **YWHAG** | **14-3-3 protein gamma** | **58.3** | **4.816** | **0.012** | **0.895** | **17** | **10** |
| Q9UBW5 | BIN2 | Bridging integrator 2 | 3.2 | -1.851 | 0.013 | 0.895 | 0 | 1 |
| Q9HD45 | TM9SF3 | Transmembrane 9 superfamily member 3 | 14.1 | 4.770 | 0.013 | 0.895 | 5 | 0 |
| **P29622** | **SERPINA4** | **Kallistatin** | **65.1** | **1.300** | **0.013** | **0.895** | **17** | **13** |
| **P47972** | **NPTX2** | **Neuronal pentraxin-2** | **43.9** | **-1.503** | **0.014** | **0.895** | **17** | **13** |
| **P17936** | **IGFBP3** | **Insulin-like growth factor-binding protein 3** | **44.7** | **1.317** | **0.016** | **0.895** | **17** | **13** |
| **P13521** | **SCG2** | **Secretogranin-2** | **52.2** | **-1.304** | **0.017** | **0.895** | **17** | **13** |
| Q16787 | LAMA3 | Laminin subunit alpha-3 | 3.6 | 1.503 | 0.017 | 0.895 | 1 | 0 |
| **P02647** | **APOA1** | **Apolipoprotein A-I** | **78.3** | **1.581** | **0.017** | **0.895** | **17** | **13** |
| **P01011** | **SERPINA3** | **Alpha-1-antichymotrypsin** | **81.1** | **1.251** | **0.019** | **0.895** | **17** | **13** |
| **P01213** | **PDYN** | **Proenkephalin-B** | **42.9** | **-8.781** | **0.019** | **0.895** | **4** | **8** |
| **P14174** | **MIF** | **Macrophage migration inhibitory factor** | **20.9** | **1.268** | **0.019** | **0.895** | **17** | **13** |
| P07195 | LDHB | L-lactate dehydrogenase B chain | 56.6 | 1.171 | 0.020 | 0.895 | 17 | 13 |
| Q15459 | SF3A1 | Splicing factor 3A subunit 1 | 8.1 | 6.508 | 0.020 | 0.895 | 13 | 5 |
| **P02763** | **ORM1** | **Alpha-1-acid glycoprotein 1** | **41.3** | **5.683** | **0.022** | **0.895** | **16** | **9** |
| **P16152** | **CBR1** | **Carbonyl reductase [NADPH] 1** | **75.1** | **1.262** | **0.022** | **0.895** | **17** | **13** |

**Supplementary Table 2.** Differentially expressed proteins in cohort 2 based on mass spectrometry data analysis (continued)

| **Uniprot Accession** | **Gene name** | **Protein name** | **Sequence coverage (%)** | **Fold change** | **p-value** | **q-value** | **# DLB samples** | **# control samples** |
| --- | --- | --- | --- | --- | --- | --- | --- | --- |
| **P12955** | **PEPD** | **Xaa-Pro dipeptidase** | **45** | **1.301** | **0.022** | **0.895** | **17** | **13** |
| **P50453** | **SERPINB9** | **Serpin B9** | **53.7** | **-5.718** | **0.023** | **0.895** | **7** | **11** |
| P51665 | PSMD7 | 26S proteasome non-ATPase regulatory subunit 7 | 7.4 | 1.515 | 0.023 | 0.895 | 1 | 0 |
| Q99832 | CCT7 | T-complex protein 1 subunit eta | 8.6 | 1.588 | 0.023 | 0.895 | 1 | 0 |
| Q16539 | MAPK14 | Mitogen-activated protein kinase 14 | 5.1 | -1.729 | 0.024 | 0.895 | 0 | 1 |
| P0C7U0 | ELFN1 | Protein ELFN1 | 4.6 | -1.978 | 0.024 | 0.895 | 0 | 1 |
| Q15818 | NPTX1 | Neuronal pentraxin-1 | 56.9 | -1.193 | 0.024 | 0.895 | 17 | 13 |
| **Q01469** | **FABP5** | **Fatty acid-binding protein, epidermal** | **80.7** | **1.239** | **0.024** | **0.895** | **17** | **13** |
| **P0C6S8** | **LINGO3** | **Leucine-rich repeat and immunoglobulin-like domain-containing nogo receptor-interacting protein 3** | **20.4** | **-3.082** | **0.025** | **0.895** | **12** | **13** |
| P10644 | PRKAR1A | cAMP-dependent protein kinase type I-alpha regulatory subunit | 36.5 | 2.061 | 0.026 | 0.895 | 1 | 0 |
| Q9HC38 | GLOD4 | Glyoxalase domain-containing protein 4 | 52.7 | 1.185 | 0.026 | 0.895 | 17 | 13 |
| **Q15262** | **PTPRK** | **Receptor-type tyrosine-protein phosphatase kappa** | **16.9** | **-1.207** | **0.026** | **0.895** | **17** | **13** |
| **Q7Z7M8** | **B3GNT8** | **UDP-GlcNAc:betaGal beta-1,3-N-acetylglucosaminyltransferase 8** | **26.7** | **4.703** | **0.028** | **0.895** | **12** | **4** |
| P10646 | TFPI | Tissue factor pathway inhibitor | 17.1 | 2.023 | 0.028 | 0.895 | 2 | 0 |
| **O15540** | **FABP7** | **Fatty acid-binding protein, brain** | **68.9** | **2.687** | **0.028** | **0.895** | **17** | **11** |
| **P30508** | **HLA-C** | **HLA class I histocompatibility antigen, Cw-12 alpha chain** | **59** | **-6.500** | **0.028** | **0.895** | **4** | **8** |
| **P01009** | **SERPINA1** | **Alpha-1-antitrypsin;Short peptide from AAT** | **72** | **2.543** | **0.029** | **0.895** | **17** | **13** |
| **Q9UHG2** | **PCSK1N** | **ProSAAS** | **68.5** | **-1.208** | **0.029** | **0.895** | **17** | **13** |
| P61278 | SST | Somatostatin | 20.7 | -2.235 | 0.029 | 0.895 | 2 | 5 |
| **Q14520** | **HABP2** | **Hyaluronan-binding protein 2** | **46.3** | **1.316** | **0.030** | **0.895** | **17** | **13** |
| **P59665** | **DEFA1** | **Neutrophil defensin 1** | **28.7** | **4.850** | **0.030** | **0.895** | **17** | **10** |
| **P02787** | **TF** | **Serotransferrin** | **83.4** | **1.799** | **0.030** | **0.895** | **17** | **13** |
| **P62937** | **PPIA** | **Peptidyl-prolyl cis-trans isomerase A** | **70.9** | **1.240** | **0.031** | **0.895** | **17** | **13** |
| Q01151 | CD83 | CD83 antigen | 4.9 | 1.590 | 0.031 | 0.895 | 1 | 0 |
| **P00558** | **PGK1** | **Phosphoglycerate kinase 1** | **77.5** | **1.256** | **0.033** | **0.895** | **17** | **13** |
| A0A0C4DH73 | IGKV1-12 | Immunoglobulin kappa variable 1-12 | 13.7 | -1.603 | 0.033 | 0.895 | 0 | 1 |
| P0DP25 | CALM3 | Calmodulin-3 | 61.1 | 1.186 | 0.033 | 0.895 | 17 | 13 |
| **Q8WY21** | **SORCS1** | **VPS10 domain-containing receptor SorCS1** | **38.1** | **-1.273** | **0.034** | **0.895** | **17** | **13** |
| **Q7Z7G0** | **ABI3BP** | **Target of Nesh-SH3** | **22.2** | **1.233** | **0.035** | **0.895** | **17** | **13** |
| CON__Q3MHN5 | |  | 19,2 | -1.465 | 0.035 | 0,895 | 0 | 1 |
| **P01008** | **SERPINC1** | **Antithrombin-III** | **71.8** | **1.239** | **0.035** | **0.895** | **17** | **13** |
| Q96GD0 | PDXP | Pyridoxal phosphate phosphatase | 28.7 | 3.596 | 0.037 | 0.895 | 8 | 2 |
| Q4LDE5 | SVEP1 | Sushi, von Willebrand factor type A, EGF and pentraxin domain-containing protein 1 | 9.5 | 3.207 | 0.037 | 0.895 | 11 | 4 |
| **P36980** | **CFHR2** | **Complement factor H-related protein 2** | **68.3** | **1.517** | **0.037** | **0.895** | **17** | **13** |
| P13497 | BMP1 | Bone morphogenetic protein 1 | 9.3 | 3.890 | 0.038 | 0.895 | 15 | 7 |
| **P01042** | **KNG1** | **Kininogen-1** | **67.9** | **1.463** | **0.039** | **0.895** | **17** | **13** |

**Supplementary Table 2.** Differentially expressed proteins in cohort 2 based on mass spectrometry data analysis (continued)

| **Uniprot Accession** | **Gene name** | **Protein name** | **Sequence coverage (%)** | **Fold change** | **p-value** | **q-value** | **# DLB samples** | **# control samples** |
| --- | --- | --- | --- | --- | --- | --- | --- | --- |
| **Q9BZ76** | **CNTNAP3** | **Contactin-associated protein-like 3** | **30.4** | **-3.497** | **0.041** | **0.895** | **9** | **11** |
| **Q08554** | **DSC1** | **Desmocollin-1** | **24.6** | **2.691** | **0.042** | **0.895** | **17** | **11** |
| **P0C0L5** | **C4B** | **Complement C4-B** | **80** | **1.219** | **0.044** | **0.895** | **17** | **13** |
| **P06744** | **GPI** | **Glucose-6-phosphate isomerase** | **44.4** | **1.250** | **0.044** | **0.895** | **17** | **13** |
| Q6ZN30 | BNC2 | Zinc finger protein basonuclin-2 | 4.3 | -21.430 | 0.044 | 0.895 | 7 | 10 |
| **P00568** | **AK1** | **Adenylate kinase isoenzyme 1** | **59.8** | **3.112** | **0.044** | **0.895** | **17** | **10** |
| **Q16853** | **AOC3** | **Membrane primary amine oxidase** | **19.8** | **-3.288** | **0.045** | **0.895** | **9** | **11** |
| **Q9UIW2** | **PLXNA1** | **Plexin-A1** | **19.5** | **-2.576** | **0.045** | **0.895** | **14** | **13** |
| Q5BIV9 | SPRN | Shadow of prion protein | 22.5 | 3.455 | 0.045 | 0.895 | 6 | 1 |
| **Q14563** | **SEMA3A** | **Semaphorin-3A** | **27.8** | **-3.326** | **0.045** | **0.895** | **9** | **11** |
| **P27348** | **YWHAQ** | **14-3-3 protein theta** | **43.7** | **3.297** | **0.045** | **0.895** | **14** | **7** |
| Q16473 | TNXA | Putative tenascin-XA | 62.4 | -1.597 | 0.046 | 0.895 | 0 | 1 |
| Q9ULB1 | NRXN1 | Neurexin-1 | 48.5 | -1.171 | 0.046 | 0.895 | 17 | 13 |
| **P31150** | **GDI1** | **Rab GDP dissociation inhibitor alpha** | **69.4** | **1.205** | **0.046** | **0.895** | **17** | **13** |
| Q9NZL9 | MAT2B | Methionine adenosyltransferase 2 subunit beta | 17.4 | 2.582 | 0.047 | 0.895 | 3 | 0 |
| Q9HDC9 | APMAP | Adipocyte plasma membrane-associated protein | 28.4 | 2.333 | 0.047 | 0.895 | 3 | 0 |
| **Q9GZQ8** | **MAP1LC3B** | **Microtubule-associated proteins 1A/1B light chain 3B** | **27.2** | **6.112** | **0.048** | **0.895** | **12** | **5** |
| P04899 | GNAI2 | Guanine nucleotide-binding protein G(i) subunit alpha-2 | 10.4 | -1.871 | 0.049 | 0.895 | 0 | 1 |
| P07307 | ASGR2 | Asialoglycoprotein receptor 2 | 33.8 | -1.695 | 0.049 | 0.895 | 0 | 1 |
| **P49862** | **KLK7** | **Kallikrein-7** | **50.2** | **-4.699** | **0.049** | **0.895** | **10** | **12** |
| P35612 | ADD2 | Beta-adducin | 6.1 | 1.375 | 0.050 | 0.895 | 1 | 0 |

*List of 93 differentially expressed proteins in CSF from patients with DLB compared to CSF from cognitively normal controls (p<0.05)*

*Proteins in bold (n=60) also fulfilled the predefined criteria for candidate biomarkers (p<0.05, fold change >1.2, >20% sequence coverage, detected in at least 50% of DLB patients or controls).*

**
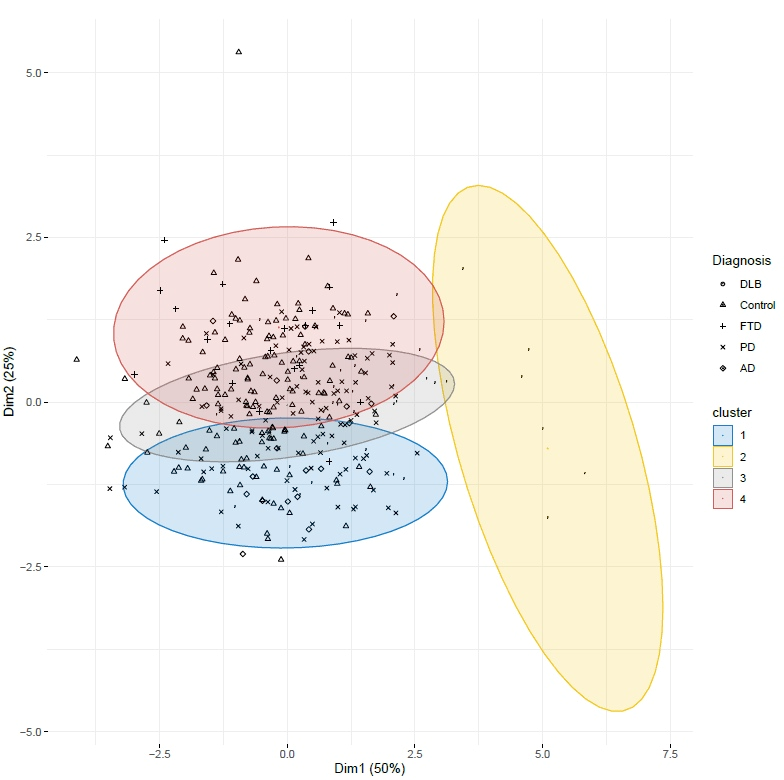
**

**Supplementary Figure 1.** K-means cluster plot of identified clusters
*Cluster solution was plotted against the first two dimensions The symbols represent the clinical diagnosis (circle = DLB; triangle = Control; plus = FTD, cross = PD, diamond = AD). The colors represent the four different clusters. Abbreviations: AD, Alzheimer’s disease; DLB, dementia with Lewy bodies; FTD, Frontotemporal dementia; PD, Parkinson’s disease.*

**Supplementary Table 3.** Comprehensive overview of CSF studies of the identified candidate biomarkers for DLB

| **Neurosceretory protein VGF (VGF)** | | | |
| --- | --- | --- | --- |
| **Study** | **Method** | **Participants** | **Results** |
| Brinkmalm (2018) [1] | Targeted Mass Spectrometry (PRM-MS) | 10 AD patients 13 healthy controls | VGF ↓ in AD vs controls |
| Carrette (2003) [2] | Quantitative Mass Spectrometry | 9 AD 10 healthy controls | VGF ↓ in AD vs controls |
| Duits (2018) [3] | Targeted Mass Spectrometry (PRM-MS) | 40 AD 40 MCI 40 non-demented controls | VGF ↓ in AD vs controls  VGF ↑ in MCI vs AD |
| Hendrikson (2015) [4] | Targeted Mass Spectrometry (SRM) | 30 AD 30 controls | VGF ↓ in AD vs controls |
| Holtta (2015) [5] | Quantitative Mass Spectrometry | 8 AD 8 non-demented controls | VGF ↓ in AD vs controls |
| Jahn (2011) [6] | Quantitative Mass Spectrometry | 34 AD 17 controls | VGF ↓ in AD vs controls |
| Llano (2017) [7] | Targeted Mass Spectrometry (MRM) | 66 AD 135 MCI 86 normal aged controls | VGF ↓ in AD vs controls |
| Selle (2005) [8] | Quantitative Mass Spectrometry | 127 AD 86 non-demented controls 66 non-AD demented patients | VGF ↓ in AD vs controls |
| Simonsen (2007) [9] | Quantitative Mass Spectrometry | 85 AD 20 FTD 32 healthy controls | VGF ↓ in AD vs controls |
| Wijte (2012) [10] | Quantitative Mass Spectrometry | 20 AD 20 non-demented controls  NB. Postmortem CSF | VGF ↓ in AD vs controls |
| Ruetschi (2012) [11] | Quantitative Mass Spectrometry | 16 FTD 12 non-demented controls | VGF_26-62_ ↓ in FTD vs controls |
| Pasinetti (2006) [12] | Quantitative Mass Spectrometry | 36 ALS 21 healthy controls | VGF_398-411_ ↓ in ALS vs controls |
| Zhao (2008) [13] | ELISA | 17 ALS 21 healthy controls | VGF_398-411_ ↓ in ALS vs controls |
| Huang (2006) [14] | Quantitative Mass Spectrometry | 41 Schizophrenia 40 healthy controls | VGF_23-62_ ↑ in schizophrenia vs controls |

**Supplementary Table 3.** Comprehensive overview of CSF studies of the identified candidate biomarkers for DLB (continued)

| **Secretogranin-2 (SCG2)** | | | |
| --- | --- | --- | --- |
| **Study** | **Method** | **Participants** | **Results** |
| Brinkmalm (2018) [1] | Targeted Mass Spectrometry (PRM-MS) | 10 AD patients 13 healthy controls | SCG2 ↓ in AD vs controls |
| Llano (2017) [7] | Targeted Mass Spectrometry (MRM) | 66 AD 86 normal aged controls | SCG2 ↓ in AD vs controls |
| Mattsson (2007) [15] | Quantitative Mass Spectrometry | 46 MS  46 healthy siblings 50 healthy controls | SCG2 ↓ in MS vs siblings and controls |
| **ProSAAS (PCSK1N)** | | | |
| **Study** | **Method** | **Participants** | **Results** |
| Abdi (2006) [16] | Quantitative Mass Spectrometry | 10 AD 10 PD 5 DLB  10 normal aged controls | ProSAAS ↓ in AD vs controls No significant changes in DLB and PD |
| Jahn (2011) [6] | Quantitative Mass Spectrometry | 34 AD 17 controls | ProSAAS ↓ in AD vs controls |
| Wang (2016) [17] | Quantitative Mass Spectrometry | 8 AD 4 non-demented controls | ProSAAS ↓ in AD vs controls |
| Davidsson (2002) [18] | Quantitative Mass Spectrometry | 15 FTD 12 non-demented controls | ProSAAS ↓ in FTD vs controls |
| **Neuronal pentraxin-2 (NPTX2) and Neuronal pentraxin receptor (NPTXR)** | | | |
| **Study** | **Method** | **Participants** | **Results** |
| Hendrikson (2015) [4] | Targeted Mass Spectrometry (SRM) | 30 AD 30 controls | NPTXR ↓ in AD vs controls |
| Llano (2017) [7] | Targeted Mass Spectrometry (MRM) | 66 AD 135 MCI 86 normal aged controls | NPTX2 and NPTXR ↓ in AD vs controls  NPTX2 and NPTXR ↓ in MCI converters vs non-converters |
| Perrin (2011) [19] | Quantitative Mass Spectrometry | 24 AD 24 non-demented controls | NPTXR ↓ in AD vs controls |
| Spellman (2015) [20] | Targeted Mass Spectrometry (MRM) | 66 AD 134 MCI 85 healthy controls | NPTX2 and NPTXR ↓ in AD vs controls |
| Xiao (2017) [21] | ELISA and Westernblot | 30 AD 36 healthy controls | NPTX2 and NPTXR ↓ in AD vs controls |

**Supplementary Table 3.** Comprehensive overview of CSF studies of the identified candidate biomarkers for DLB (continued)

| **Proenkephalin-B (PDYN)** | | | |
| --- | --- | --- | --- |
| **Study** | **Method** | **Participants** | **Results** |
| Llano (2017) [7] | Targeted Mass Spectrometry (MRM) | 66 AD  135 MCI 86 normal aged controls | PDYN ↓ in AD vs controls |

*Table lists CSF studies of the six identified candidate biomarkers for DLB in neurological and psychiatric diseases.****Abbreviations****: AD, Alzheimer’s disease; ALS, Amyotrophic lateral sclerosis; DLB, dementia with Lewy bodies; FTD, Frontotemporal dementia; MRM, Multiple reaction monitoring; MS, Multiple sclerosis; NPTX2, Neuronal pentraxin 2; NPTXR, Neuronal pentraxin receptor, PCSK1N, PRM-MS, Parallel Reaction Monitoring Mass Spectrometry; ProSAAS; PDYN, Proenkephalin-B; SCG2, Scretogranin-2 ;SRM, Selected Reaction Monitoring; VGF, Neurosecretory protein VGF.*

**References Supplementary Table 3**

1. Brinkmalm G, Sjodin S, Simonsen AH, Hasselbalch SG, Zetterberg H, Brinkmalm A, et al. A Parallel Reaction Monitoring Mass Spectrometric Method for Analysis of Potential CSF Biomarkers for Alzheimer's Disease. Proteomics Clin Appl. 2018;12(1).

2. Carrette O, Demalte I, Scherl A, Yalkinoglu O, Corthals G, Burkhard P, et al. A panel of cerebrospinal fluid potential biomarkers for the diagnosis of Alzheimer's disease. Proteomics. 2003;3(8):1486-94.

3. Duits FH, Brinkmalm G, Teunissen CE, Brinkmalm A, Scheltens P, Van der Flier WM, et al. Synaptic proteins in CSF as potential novel biomarkers for prognosis in prodromal Alzheimer's disease. Alzheimers Res Ther. 2018;10(1):5.

4. Hendrickson RC, Lee AY, Song Q, Liaw A, Wiener M, Paweletz CP, et al. High Resolution Discovery Proteomics Reveals Candidate Disease Progression Markers of Alzheimer's Disease in Human Cerebrospinal Fluid. PLoS One. 2015;10(8):e0135365.

5. Holtta M, Minthon L, Hansson O, Holmen-Larsson J, Pike I, Ward M, et al. An integrated workflow for multiplex CSF proteomics and peptidomics-identification of candidate cerebrospinal fluid biomarkers of Alzheimer's disease. J Proteome Res. 2015;14(2):654-63.

6. Jahn H, Wittke S, Zurbig P, Raedler TJ, Arlt S, Kellmann M, et al. Peptide fingerprinting of Alzheimer's disease in cerebrospinal fluid: identification and prospective evaluation of new synaptic biomarkers. PLoS One. 2011;6(10):e26540.

7. Llano DA, Bundela S, Mudar RA, Devanarayan V, Alzheimer's Disease Neuroimaging I. A multivariate predictive modeling approach reveals a novel CSF peptide signature for both Alzheimer's Disease state classification and for predicting future disease progression. PLoS One. 2017;12(8):e0182098.

8. Selle H, Lamerz J, Buerger K, Dessauer A, Hager K, Hampel H, et al. Identification of novel biomarker candidates by differential peptidomics analysis of cerebrospinal fluid in Alzheimer's disease. Comb Chem High Throughput Screen. 2005;8(8):801-6.

9. Simonsen AH, McGuire J, Podust VN, Hagnelius NO, Nilsson TK, Kapaki E, et al. A novel panel of cerebrospinal fluid biomarkers for the differential diagnosis of Alzheimer's disease versus normal aging and frontotemporal dementia. Dement Geriatr Cogn Disord. 2007;24(6):434-40.

10. Wijte D, McDonnell LA, Balog CI, Bossers K, Deelder AM, Swaab DF, et al. A novel peptidomics approach to detect markers of Alzheimer's disease in cerebrospinal fluid. Methods. 2012;56(4):500-7.

11. Ruetschi U, Zetterberg H, Podust VN, Gottfries J, Li S, Hviid Simonsen A, et al. Identification of CSF biomarkers for frontotemporal dementia using SELDI-TOF. Exp Neurol. 2005;196(2):273-81.

12. Pasinetti GM, Ungar LH, Lange DJ, Yemul S, Deng H, Yuan X, et al. Identification of potential CSF biomarkers in ALS. Neurology. 2006;66(8):1218-22.

13. Zhao Z, Lange DJ, Ho L, Bonini S, Shao B, Salton SR, et al. Vgf is a novel biomarker associated with muscle weakness in amyotrophic lateral sclerosis (ALS), with a potential role in disease pathogenesis. Int J Med Sci. 2008;5(2):92-9.

14. Huang JT, Leweke FM, Oxley D, Wang L, Harris N, Koethe D, et al. Disease biomarkers in cerebrospinal fluid of patients with first-onset psychosis. PLoS Med. 2006;3(11):e428.

15. Mattsson N, Ruetschi U, Podust VN, Stridsberg M, Li S, Andersen O, et al. Cerebrospinal fluid concentrations of peptides derived from chromogranin B and secretogranin II are decreased in multiple sclerosis. J Neurochem. 2007;103(5):1932-9.

16. Abdi F, Quinn JF, Jankovic J, McIntosh M, Leverenz JB, Peskind E, et al. Detection of biomarkers with a multiplex quantitative proteomic platform in cerebrospinal fluid of patients with neurodegenerative disorders. J Alzheimers Dis. 2006;9(3):293-348.

17. Wang J, Cunningham R, Zetterberg H, Asthana S, Carlsson C, Okonkwo O, et al. Label-free quantitative comparison of cerebrospinal fluid glycoproteins and endogenous peptides in subjects with Alzheimer's disease, mild cognitive impairment, and healthy individuals. Proteomics Clin Appl. 2016;10(12):1225-41.

18. Davidsson P, Sjogren M, Andreasen N, Lindbjer M, Nilsson CL, Westman-Brinkmalm A, et al. Studies of the pathophysiological mechanisms in frontotemporal dementia by proteome analysis of CSF proteins. Brain Res Mol Brain Res. 2002;109(1-2):128-33.

19. Perrin RJ, Craig-Schapiro R, Malone JP, Shah AR, Gilmore P, Davis AE, et al. Identification and validation of novel cerebrospinal fluid biomarkers for staging early Alzheimer's disease. PLoS One. 2011;6(1):e16032.

20. Spellman DS, Wildsmith KR, Honigberg LA, Tuefferd M, Baker D, Raghavan N, et al. Development and evaluation of a multiplexed mass spectrometry based assay for measuring candidate peptide biomarkers in Alzheimer's Disease Neuroimaging Initiative (ADNI) CSF. Proteomics Clin Appl. 2015;9(7-8):715-31.

21. Xiao MF, Xu D, Craig MT, Pelkey KA, Chien CC, Shi Y, et al. NPTX2 and cognitive dysfunction in Alzheimer's Disease. Elife. 2017;6.
